# LncRNA FLJ20021 Restrains Fibroblast Senescence and Matrix Remodeling in Pelvic Organ Prolapse by Promoting XRCC6‐Dependent ADI1 Transcription

**DOI:** 10.1002/advs.78346

**Published:** 2026-10-25

**Authors:** YanFeng Yang, BingJie Rui, GuangHai Rui, YiNan Duan, Ji Hyang Kim

**Affiliations:** ^1^ Department of Obstetrics and Gynecology Fushun Maternal and Child Health Hospital Fushun China; ^2^ Department of Obstetrics and Gynecology Shengjing Hospital of China Medical University Shenyang China; ^3^ Department of Obstetrics and Gynecology CHA University School of Medicine Pocheon Republic of Korea; ^4^ Department of Obstetrics and Gynecology Fushun Second Hospital Fushun China; ^5^ Department of Gynecology Liaoning Provincial Maternal and Child Health Hospital Shenyang Liaoning China; ^6^ Fertility Center CHA University Bundang Medical Center CHA University Seongnam Republic of Korea

**Keywords:** extracellular matrix, long non‐coding RNA, uterosacral ligament fibroblasts

## Abstract

Pelvic‐organ prolapse (POP) is an age‐associated disorder that is often accompanied by uterosacral ligament (USL) weakening and extracellular matrix (ECM) degradation; however, the upstream regulators linking fibroblast aging to ECM remodeling remain incompletely defined. Here, we identify an uncharacterized long non‐coding RNA (lncRNA), FLJ20021, as significantly downregulated in POP‐derived USL tissues and primary human uterosacral‐ligament fibroblasts (hUSLFs) compared with non‐POP controls. FLJ20021 depletion reduces hUSLF proliferation and increases apoptosis and senescence, whereas FLJ20021 overexpression reverses these effects and shifts ECM‐related markers toward a matrix‐preserving profile. Mechanistically, FLJ20021 directly binds the DNA‐binding domain (DBD) of X‐ray repair cross‐complementing 6 (XRCC6), facilitates XRCC6 occupancy at the ADI1 promoter, and promotes XRCC6‐dependent ADI1 transcription. Gain‐ and loss‐of‐function as well as rescue experiments place XRCC6 downstream of FLJ20021 and support ADI1 as a required effector for FLJ20021‐dependent cellular and ECM‐related phenotypes. In POP‐relevant murine injury models, XRCC6 and ADI1 are coordinately reduced with ECM‐marker imbalance, and FLJ20021 overexpression partially restores ADI1 and ECM‐related readouts in vivo. Together, these data support a model in which FLJ20021 interacts with XRCC6 to regulate ADI1 expression, thereby linking fibroblast‐fate control to ECM homeostasis and providing mechanistic insight into POP‐associated tissue degeneration.

## Graphical abstract

FLJ20021 interacts with XRCC6 to regulate ADI1 transcription and downstream cellular phenotypes in hUSLFs. Reduced FLJ20021 in POP is associated with lower ADI1, increased apoptosis, senescence, and an ECM‐degrading marker profile. Conversely, FLJ20021 overexpression enhances XRCC6‐dependent ADI1 expression, reduces apoptosis and senescence, and shifts ECM‐related markers toward a more homeostatic state. This figure was created with FigDraw.

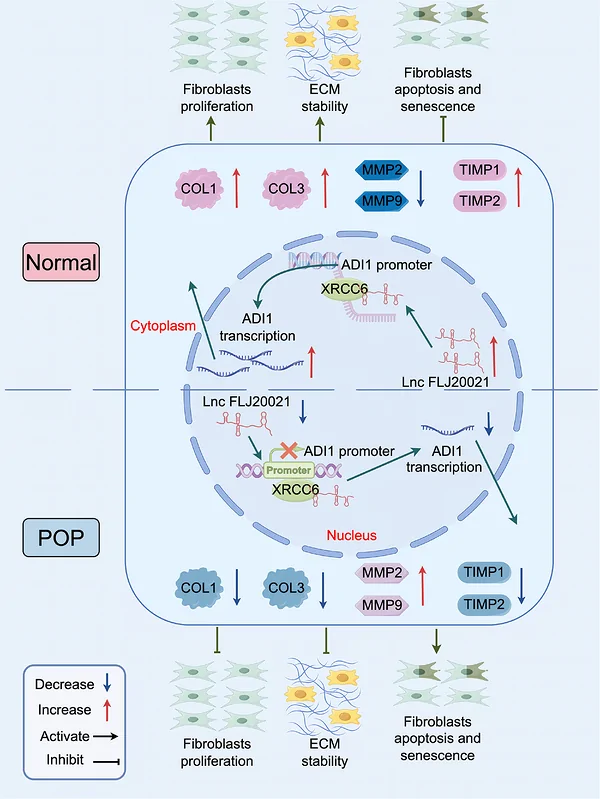

**Abbreviations**
ADI1: Acireductone dioxygenase 1
ChIP: Chromatin immunoprecipitation
ChIP–qPCR: Chromatin immunoprecipitation‐qPCR
COLI: Collagen type I
COLIII: Collagen type III
DBD: DNA‐binding core domain
DSB: DNA double‐strand break
ECM: Extracellular matrix
FBS: Fetal bovine serum
FISH: Fluorescence in situ hybridization
GH: Genital hiatus
hUSLFs: Human uterosacral ligament fibroblasts
IF: Immunofluorescence
ISH: In situ hybridization
lncRNAs: Long non‐coding RNAs
MMPs: Matrix metalloproteinases
MS: Mass spectrometry
MT1‐MMP: membrane‐type 1 matrix metalloproteinase
OVX: Ovariectomy
POP: Pelvic organ prolapse
RBPs: RNA‐binding proteins
RIP: RNA immunoprecipitation
RNA‐seq: RNA sequencing
RT: Room temperature
RT‐qPCR: Reverse Transcription Quantitative Polymerase Chain Reaction
SASP: Senescence‐associated secretory phenotype
TIMPs: Tissue inhibitors of metalloproteinases
USL: Uterosacral ligament
VD: vaginal distension
WB: Western blotting
XRCC6: X‐ray repair cross‐complementing 6

## 1 Introduction

POP is a prevalent degenerative gynecologic condition characterized by the descent of pelvic organs due to failure of the supportive pelvic floor structures [1]. Its incidence rises with age, and the lifetime risk of requiring surgical intervention for POP is approximately 10% [2]. POP is considered a multifactorial disorder with several independent risk factors, including advancing age, multiparity (especially vaginal childbirth), obesity‐related chronically elevated intra‐abdominal pressure, and connective tissue disorders such as Ehlers‐Danlos syndrome that compromise collagen integrity [3, 4, 5]. These factors contribute to weakening of the pelvic support complex, which consists of the levator ani muscles, endopelvic fascia, and uterosacral/cardinal ligaments, ultimately leading to organ prolapse. POP is increasingly recognized as an age‐related degenerative disease, and recent studies implicate cellular aging processes in its pathogenesis [1]. In particular, fibroblasts within pelvic support tissues show features of cellular senescence in older women with POP, suggesting that senescent fibroblasts with their diminished regenerative capacity and altered secretory profiles contribute to the loss of connective‐tissue support that underlies prolapse [2]. Beyond chronological aging, endocrine, mechanical, and inflammatory cues converge on pelvic‐support fibroblasts to modulate ECM turnover. In ovariectomized primates, estradiol increases collagen type I/III(COL I/III) mRNA levels in vaginal connective‐tissue fibroblasts, suggesting endocrine control of fibroblast‐mediated ECM synthesis [6]. Experimental stretch of hUSLFs using a Flexcell system induces cell elongation with F‐actin rearrangement and ECM remodeling (directional changes in COL I/III and MMP1), highlighting a mechanotransduction route linking pelvic loading to fibroblast dysfunction [7]. Studies also indicate that POP is associated with increased pro‐inflammatory cytokines (e.g., IL‐1β, TNF‐α, IFN‐γ) in the anterior vaginal wall, creating a microenvironment that alters collagen metabolism and the MMP/TIMP balance [8]. Together, these observations offer a plausible mechanistic framework linking established risk factors (e.g., age and childbirth) to molecular and structural degeneration of pelvic support tissues.

Advances in whole‐transcriptome sequencing have opened new avenues to investigate such mechanisms by profiling non‐coding RNA changes in pelvic floor disorders. High‐throughput RNA sequencing of pelvic support tissues has identified numerous differentially expressed genes and non‐coding RNAs in POP, highlighting the potential regulatory roles of lncRNAs in disease development [9]. LncRNAs are >200‐nucleotide transcripts lacking protein‐coding potential that regulate gene expression at multiple levels. They often function through complex interactions with DNA, other RNAs, and RNA‐binding proteins (RBPs) to influence chromatin states, mRNA stability, or translation [10]. For example, lncRNA JPX functions as an enhancer RNA in regulating vascular smooth muscle cell senescence by acting as a scaffold that integrates phosphorylated p65/RelA and bromodomain‐containing protein 4 into a chromatin remodeling complex, thereby promoting SASP gene transcription [11]. In fibroblasts and other cell types, lncRNA–RBP complexes can modulate signaling pathways related to senescence and ECM turnover. For example, in human fibroblasts, lncRNA MAGI2‐AS3 interacted with RBPs HSPA8 and stabilized HSPA8 protein, thereby reducing intracellular H_2_O_2_ levels and delaying cell senescence via inhibition of the ROS/MAP2K6/p38 signaling pathway [12]. Other lncRNAs, such as ANRIL, scaffold chromatin‐modifying complexes to epigenetically silence or activate senescence‐associated genes [13]. These findings underscore that lncRNAs are critical orchestrators of gene networks in aging cells. Transcriptome analyses in POP have begun to uncover lncRNAs dysregulated in uterosacral ligament fibroblasts [9], raising the possibility that non‐coding RNAs contribute to pelvic floor tissue dysfunction. Notably, many POP‐associated lncRNAs appear enriched in “protein binding” functions, suggesting that they may exert effects via interactions with RBPs [9]. Understanding how lncRNAs interface with RNA‐binding proteins in pelvic fibroblasts could reveal novel regulatory pathways driving senescence and matrix remodeling in POP.

USLs and related connective‐tissue supports are central to maintaining normal pelvic organ position. hUSLFs synthesize and remodel the ECM, including COL I and COL III, elastin, fibronectin, and proteoglycans, which collectively confer tensile strength and elasticity to the pelvic floor [14, 15]. In POP, accumulating evidence indicates that hUSLFs become dysfunctional, leading to aberrant ECM remodeling and weakened support. Structurally, prolapsed tissues contain less overall collagen but exhibit a shift in collagen composition and turnover: there is an increased COLI/COLIII ratio that may promote defective collagen fibril assembly and heightened fibril stiffness, along with elevated levels of matrix metalloproteinases such as MMP9 [16]. Fibroblasts from prolapsed ligaments show increased turnover of collagen and accumulation of “poor‐quality” collagen fibers that lack normal tensile strength. An imbalance between matrix metalloproteinases (MMPs) and their tissue inhibitors of metalloproteinases (TIMPs) has been observed in POP fibroblasts, with excess protease activity (MMP2 and MMP9 upregulation) and reduced TIMP1 and TIMP2 expression [17, 18]. This imbalance accelerates collagen degradation beyond its synthesis, resulting in deficient and disorganized connective tissue. Senescence of fibroblasts is thought to exacerbate an inflammatory and matrix‐degrading secretory phenotype. Senescent cells secrete cytokines, chemokines, and proteases (a senescence‐associated secretory phenotype, SASP) that can further degrade collagen and disrupt the local ECM architecture [19, 20, 21]. Although cellular senescence is characterized by an essentially irreversible cell cycle arrest, clearance of senescent cells using “senolytic” drugs has been shown to reduce their accumulation, alleviate age‐related symptoms and pathologies [22, 23]. Therefore, elucidating the mechanisms underlying decreased fibroblast proliferation, enhanced cellular senescence, and ECM degradation in POP is important for understanding POP pathophysiology and may help to inform future approaches to POP management.

Given the paucity of mechanistic studies on lncRNAs in POP, we focused on the uncharacterized lncRNA FLJ20021 located on chromosome 4q (NC_000004.12) [24]. We hypothesized that decreased FLJ20021 expression may contribute to POP‐associated fibroblast dysfunction by promoting cellular senescence and disrupting ECM homeostasis in hUSLFs. Therefore, this study aimed to (i) characterize FLJ20021 expression patterns in USL tissues and primary hUSLFs, (ii) determine the functional consequences of FLJ20021 gain‐ and loss‐of‐function on fibroblast proliferation, apoptosis, senescence, and ECM‐related readouts, and (iii) elucidate the underlying molecular mechanism by identifying FLJ20021‐interacting proteins and downstream transcriptional effectors. Collectively, our work seeks to provide a testable mechanistic framework linking a POP‐dysregulated lncRNA to hUSLFs senescence and ECM remodeling, thereby offering molecular insights relevant to POP pathophysiology.

## 2 Materials and Methods

### 2.1 Human Sample Collection

USL tissue samples were collected from patients at the Department of Gynecology at Shengjing Hospital, China Medical University (Shenyang, China) in 2024. The study protocol was approved by the Institutional Ethics Committee of Shengjing Hospital (approval number: 2024PS020K). Written informed consent for sample collection was obtained from all participants. All procedures involving human participants were conducted in accordance with relevant ethical regulations. The POP group included patients diagnosed with POP‐Q stages III–IV who underwent hysterectomy, while the non‐POP group consisted of patients undergoing total hysterectomy for benign gynecological conditions. The exclusion criteria included: endometriosis, connective tissue disorders, autoimmune diseases, hormone therapy, gynecological malignancies, prior pelvic radiation, pelvic inflammatory disease, or incomplete records. Baseline clinical characteristics of the POP and non‑POP cohorts are summarized in Table S1. All patients were postmenopausal and non‐smokers. USL tissues (approximately 0.5–1 cm^3^) were rinsed in sterile PBS to remove blood and immediately divided into different portions following surgical excision: one portion was snap‐frozen in liquid nitrogen and stored at −80°C for subsequent RNA extraction; a second portion was processed immediately for primary fibroblast isolation; and the remaining portion was fixed and prepared for in situ hybridization(ISH) experiments.

### 2.2 Primary hUSLFs Isolation and Culture

Primary hUSLFs were isolated from USL tissue using an explant adherence method. Fresh uterosacral ligament tissues from the clinical samples were dissected into 1 × 1 × 1 mm pieces under sterile conditions, distributed, and subsequently cultured in Dulbecco's modified Eagle's medium (DMEM; Gibco, Carlsbad, CA, USA) high glucose medium supplemented with 10% fetal bovine serum (FBS, Gibco) and 1% streptomycin/penicillin/amphotericin B (S/P/B, Servicebio, Wuhan, China), under a humidified atmosphere containing 5% CO_2_ at 37°C. After 7–10 days, primary hUSLFs (passage 0) were grown from the small pieces. Cells at passages 2–6 were selected for all experiments described to ensure consistent cell phenotype and avoid replicative senescence.

### 2.3 Fibroblast Identification

#### 2.3.1 hUSLFs Identification

hUSLFs cultured on coverslips were fixed with 4% paraformaldehyde, permeabilized with 0.1% Triton X‐100, and blocked with 3% BSA (or 10% donkey serum for goat‐derived antibodies) at room temperature (RT). The cells were then incubated overnight at 4°C with primary antibodies against vimentin (a mesenchymal fibroblast marker), desmin (a smooth muscle marker), and pan‐cytokeratin (an epithelial cell marker). After washing, cells were incubated with appropriate fluorophore‐conjugated secondary antibodies. Stained cells were visualized under a fluorescence microscope (Nikon Eclipse C1). Primary antibodies were supplied by Wuhan Servicebio Technology Co., Ltd., including Vimentin (1:200, GB11192), Desmin (1:500, GB12075), and Pancytokeratin (1:500, GB122053). Fibroblasts were verified by the presence of strong vimentin staining in the cytoplasm, with no detectable desmin or cytokeratin signal, confirming a fibroblast‐enriched population with no myocyte or epithelial contamination.

### 2.4 Transcriptome Analysis

RNA‐seq on USL tissues from POP patients and non‐POP patients was performed to screen the differentially expressed lncRNAs, as previously described by our team [24]. Furthermore, to investigate the downstream regulatory mechanisms of FLJ20021 and XRCC6, transcriptome sequencing was conducted in POP‐relevant hUSLFs after siRNA‐mediated knockdown of FLJ20021 or XRCC6, with three biological replicates per group. Libraries were sequenced as 150‐bp paired‐end reads on the Illumina HiSeq platform (Illumina, San Diego, CA, USA). Raw reads were assessed by FastQC and then filtered with Trimmomatic (v0.36) to remove reads containing N bases, trim Illumina adapters, remove low‐quality bases (Q < 20) from both ends, perform sliding‐window trimming (window size = 5 bp), and discard reads shorter than 35 nt. Clean reads were aligned to the human reference genome using HISAT2 (v2.1.0), and mapping statistics were summarized using RSeQC (v2.6.1). Gene expression quantification was performed using StringTie (v1.3.3b) to generate raw read counts and normalized expression values (TPM/FPKM). Differential expression analysis for replicated samples was performed using DESeq2 based on raw read counts. For downstream analyses, genes with adjusted P‐value (FDR)< 0.05 and |log2 fold change| > 1 were considered differentially expressed. Sequencing library preparation and subsequent high‐throughput RNA sequencing were performed by Sangon Biotech (Shanghai, China).

### 2.5 RNA Extraction and RT‐qPCR

Total RNA was extracted from USL tissue samples and cultured hUSLFs using TRIzol Reagent (Invitrogen, Carlsbad, CA, USA) according to the manufacturer's instructions. Briefly, after lysing the cells with TRIzol, chloroform was added, and the mixture was vortexed, followed by incubation on ice. The samples were then centrifuged, and the clear aqueous phase containing RNA was carefully transferred to a new tube. RNA was precipitated by adding isopropanol and incubating. The resulting RNA pellet was washed with 75% ethanol, briefly dried, and finally resuspended in nuclease‐free water for subsequent analyses. The concentration and quality of the RNA were measured with a NanoDrop One Spectrophotometer (Thermo Fisher Scientific, CA, USA). In order to prepare for quantitative PCR analysis, complementary DNA (cDNA) was synthesized using the PrimeScript RT Reagent Kit (Takara Bio, Japan). RT–qPCR was performed on an Applied Biosystems QuantStudio 3 Real‐Time PCR System (Thermo Fisher Scientific, USA) using TB Green Premix Ex TaqII (Tli RNaseH Plus) (Takara Bio). β‐actin or GAPDH was used as the internal control for each sample, and the relative gene expression was analyzed using the 2^−ΔΔ^ ^CT^ method. The sequences of the primers were provided in Table 1.

**TABLE 1:** The sequence of primer, probe, and siRNA used in this study.

Gene	Sequence (5’‐3’)	Application
FLJ20021‐F	AAAAGCGGGTCTCCGTCTAC	RT‐qPCR
FLJ20021‐R	AACCACGTTGCCAGTCCTTG	
XRCC6‐F	CGATAATGAAGGTTCTGGAAG	
XRCC6‐R	CTGGAAGTGCTTGGTGAG	
ADI1‐F	AGTTAAGCTTATGGTGCAGGCCTGGTATAT	
ADI1‐R	TGCAGAATTCTTAGGCGGTCTGTGCCAGAA	
ADI1#1‐F	TCGGCCGCGCTTTATCTTAT	
ADI1#1‐R	TTTGCTTCCGAAAACAGCGG	
ADI1#2‐F	TCGGCCGCGCTTTATCTTAT	
ADI1#2‐R	GAATGCCGACACTGCAAAAGT	
ADI1#3‐F	CTGGGATCCGAGCTGTGAAG	
ADI1#3‐R	GTGAAGCTCACCGTAGGCAT	
β‐ACTIN‐F	GGGAAATCGTGCGTGACATTAAG	
β‐ACTIN‐R	TGTGTTGGCGTACAGGTCTTTG	
GAPDH‐F	ATCACTGCCACCCAGAAGAC	
GAPDH‐R	TTTCTAGACGGCAGGTCAGG	
U6‐F	CTCGCTTCGGCAGCACA	
U6‐R	AACGCTTCACGAATTTGCGT	
COL1‐F	GTGCGATGACGTGATCTGTGA	
COL1‐R	CGGTGGTTTCTTGGTCGGT	
COL3‐F	GGAGCTGGCTACTTCTCGC	
COL3‐R	GGGAACATCCTCCTTCAACAG	
MMP9‐F	TGTACCGCTATGGTTACACTCG	
MMP9‐R	GGCAGGGACAGTTGCTTCT	
TIMP1‐F	CTTCTGCAATTCCGACCTCGT	
TIMP1‐R	ACGCTGGTATAAGGTGGTCTG	
ELN‐F	TGCTAGAGCGAATCCAGCTT	
ELN‐R	CGCTGGTGCTCTTATCTTCC	
NRF2‐F	AGGACATGGAGCAAGTTTGG
NRF2‐R	TCTGTCAGTGTGGCTTCTGG
SOD1‐F	GAGCAGAAGGAAAGTAATGG
SOD1‐R	TACACCACAAGCCAAACGAC
si‐FLJ20021#1	GCAATAGTGGCCCAAATGT	siRNA
si‐FLJ20021#2	TGCGCCACAGAGTCTGAAA	
si‐FLJ20021#3	GACAGTTGACACCCATCTT	
si‐NC	ACGTGCCACATGGTCGACGT	
si‐XRCC6#1	CCUACGAAUUGGAGAUGAATT	
si‐XRCC6#2	GCAAGAUCGAGGUGGAGAATT	
si‐XRCC6#3	GCACAGGAGGAAAGAGCAUTT	
si‐ADI1#1	GAACTACTCCTGGATGGACAT	
si‐ADI1#2	GAGCATTTGCACTTGGACGAT	
si‐ADI1#3	ACTAACACGTGCCTCGTAAAG	
FLJ20021 sense F	taatacgactcactatagggttttcttccagctgccgga	RNA pull‐down
FLJ20021 sense R	TTTTTTTTTTTTTTTTTTGAAGGGTGAGAAATT	
FLJ20021 antisense F	taatacgactcactatagggTTTTTTTTTTTTTTTTTTGAAGGGTGAGAAATT	
FLJ20021 antisense R	ttttcttccagctgccgga	
FLJ20021 probe	CTCCCATGTTTCTCCCGTCT–3′‐DIG	FISH

### 2.6 Agarose Gel Electrophoresis

PCR products or obtaining RNA samples were mixed with loading buffer and loaded into a 2% agarose gel prepared with 1×TAE buffer and DNA stain. Electrophoresis was run at 100 V for 40 min. Finally, the bands were visualized under UV light and compared to a molecular weight marker.

### 2.7 ISH

The sections with a thickness of 3 µm were deparaffinized in xylene and rehydrated through a graded ethanol series. Prehybridization was performed in a humidified chamber at 37°C for 2 h using the appropriate prehybridization solution. Subsequently, the sections were hybridized overnight at 37°C with a DIG‐labeled FLJ20021 probe (Wuhan, BOSTER Biological Technology Co., Ltd.). After hybridization, sections were washed and incubated with anti‐digoxigenin antibody at 4°C overnight, followed by staining with a DAB substrate kit (DA1010; Solarbio, Beijing, China). The FLJ20021 probe comprised the following sequences:5’‐CCGAGGAGACGGGAGAAACATGGGAGAGGGTGGCCTTTTC‐3’. For quantitative analysis, five random fields per section from three individual sections were imaged using a microscope (Olympus IX51) with identical acquisition settings across groups. Signal intensity was quantified using ImageJ.

### 2.8 Immunohistochemistry (IHC)

Tissue slices were incubated overnight with primary antibodies at 4°C. The antibodies included XRCC6 (1:500, Proteintech), ADI1 (1:200, Proteintech), COLI (1:100, Zenbio), MMP9(1:100, Zenbio), and P21(1:5000, Proteintech). The slices were then incubated with HRP‐conjugated secondary antibodies at RT for 1 h, followed by visualization with a DAB substrate kit. Five random non‐overlapping fields were captured from each of three sections per USL sample under identical acquisition settings, and staining was quantified in ImageJ.

### 2.9 Masson's Trichrome (MT) Staining

Collagen organization in mouse USL tissues was assessed histologically using Masson's trichrome staining. Briefly, USL tissues were fixed in 4% paraformaldehyde, embedded in paraffin, and sectioned at a thickness of 5 µm using a microtome. Following deparaffinization and rehydration, the sections were stained using a Masson's trichrome staining kit according to the manufacturer's instructions. Slices were then imaged using a microscope (Olympus).

### 2.10 FISH and Immunofluorescence (IF) Staining

The slides of hUSLFs were fixed with 4% paraformaldehyde and permeabilized with 0.2% Triton X‐100. After brief digestion with proteinase K (5 µg/mL, 5 min at 37°C) and prehybridization at 37°C, the coverslips were incubated overnight at 40°C with a DIG‐labeled FLJ20021 probe (Genecreate Biological Engineering Co., Ltd) diluted in hybridization buffer. Post‐hybridization washes were performed at 37°C using decreasing concentrations of SSC. Signal detection was achieved by incubating with anti‐DIG‐HRP (1:100 in PBS) followed by tyramide signal amplification using a CY3‐tyramide reagent for 30 min in the dark. After a second blocking step with 5% BSA, the coverslips were incubated overnight at 4°C with primary anti‐XRCC6 antibody (1:500 in PBS), then with a FITC‐conjugated secondary antibody (1:100). Finally, cells were counterstained with DAPI, mounted with an anti‐fade reagent, and visualized using confocal microscopy to assess the co‐localization of FLJ20021 (red) and XRCC6 (green) in the nucleus.

### 2.11 Subcellular Fractionation

Nuclear and cytoplasmic RNA fractionations were performed using a cell fractionation kit (PARIS kit, Thermo Fisher Scientific) following the manufacturer's instruction, analyzed by RT‐qPCR successively, GAPDH mRNA and U6 small nuclear RNA were used as the cytoplasmic and nuclear endogenous control, respectively.

### 2.12 LncRNA Pull‐Down and MS

RNA pull‐down assays were performed using the RNA‐Protein Pull Down Kit (Genecreate Biological Engineering Co., Ltd.) according to the manufacturer's instructions. Biotin‐labeled RNA probes targeting FLJ20021 and a negative control (NC) were synthesized by Shanghai Sangon Biotech Co., Ltd. (Shanghai, China) (see Table 1 for probe sequences). The target RNA was in vitro transcribed using the TranscriptAid T7 High Yield Transcription Kit (Thermo Fisher Scientific) and subsequently biotinylated with the RNA 3’ End Biotinylation Kit (Thermo Fisher Scientific). The biotinylated RNA was then incubated with streptavidin‐coated magnetic beads (Thermo Fisher Scientific) to facilitate efficient binding. Following extensive washing, the RNA‐bound beads were incubated with hUSLFs lysates in lysis buffer for RNA‐protein interactions. The FLJ20021 RNA‐protein complexes were eluted and analyzed by silver staining for visualization and by MS for protein identification (performed by Genecreate Biological Engineering Co., Ltd.). All procedures were conducted under RNase‐free conditions to prevent RNA degradation.

### 2.13 RIP

According to the manufacturer's instructions, RNA‐binding protein immunoprecipitation (RIP) Kit (Genecreate Biological Engineering Co., Ltd.) was used for RIP detection. Briefly, cell lysates were prepared in RIP lysis buffer containing RNase inhibitor and protease inhibitors. The lysates were divided into equal aliquots and incubated overnight at 4°C with magnetic beads conjugated to target‐specific antibodies or control IgG. After washing with RIP wash buffer, the immunocomplexes bound to the beads were used for RNA isolation. The purified RNA was reverse transcribed into cDNA and analyzed by RT‐qPCR, with agarose gel electrophoresis confirming the specificity of the RT‐qPCR products.

### 2.14 ChIP–qPCR

The ChIP assay was conducted using the ChIP Assay Kit (Beyotime, Shanghai, China) according to the manufacturer's instructions. Briefly, hUSLFs were cross‐linked with 1% formaldehyde, collected, lysed, and sonicated to generate DNA fragments of approximately 200–1000 bp. The fragmented chromatin was then incubated overnight with an anti‐XRCC6 antibody (Proteintech, China), while normal IgG (Beyotime, China) was used as a negative control. The chromatin samples were subsequently incubated with Protein A/G magnetic beads–salmon sperm DNA for 1 h at 4°C with rotation to capture the immune complexes. After washing and elution, the cross‐links were reversed, and the DNA was purified for quantitative PCR analysis.

### 2.15 Western Blotting (WB)

Cellular total protein was extracted using protein lysis buffer. Equal amounts of protein were resolved by SDS–PAGE and electro‐transferred onto a 0.22 µm pore‐size PVDF membrane (Millipore, Billerica, MA, USA). After blocking with 5% non‐fat milk at RT for 1 h, the membrane was incubated overnight at 4°C with the primary antibody. Following washes with TBST, the membrane was incubated with appropriate secondary antibodies (Table 2) at RT for 1 h. Protein bands were visualized using an enhanced chemiluminescence (ECL) detection system.

**TABLE 2:** Antibodies used in this study.

Product	Source	No. of catalogue	Dilutions	Application
Anti‐beta Actin	Servicebio	GB15003‐100	1:1000	Western blot Primary antibody
Anti‐XRCC6	Proteintech	10723‐1‐AP	1:6000	
Anti‐ADI1	Proteintech	15133‐1‐AP	1:1000	
Anti‐Flag	Proteintech	66008‐4‐LG	1:5000	
Anti‐COLIA1	Zenbio	R26615	1:1000	
Anti‐COLIIIA1	Zenbio	R23957	1:1000	
Anti‐MMP2	Zenbio	R24993	1:1000	
Anti‐MMP9	Zenbio	R380831	1:1000	
Anti‐TIMP1	Zenbio	340983	1:1000	
Anti‐TIMP2	Zenbio	R381741	1:1000	
HRP‐Goat Anti‐Mouse	Proteintech	SA00001‐1	1:10000	Western blot secondary antibody
HRP‐Goat Anti‐Rabbit	Proteintech	SA00001‐2	1:10000	
Anti‐XRCC6	Proteintech	10723‐1‐AP	1:500	IF
Anti‐ADI1 Anti‐P21 Anti‐COLIA1 Anti‐MMP9	Proteintech Proteintech Zenbio Zenbio	15133‐1‐AP 10355‐1‐AP R26615 R380831	1:200 1:5000 1:100 1:100	IHC
Anti‐XRCC6	Proteintech	10723‐1‐AP	1:500	
488‐conjugated Goat anti‐Rabbit	Proteintech	SA00013‐2	1:500	IF secondary antibody
594‐conjugated Goat anti‐Mouse	Proteintech	SA00013‐3	1:500	

### 2.16 Cell Transfection

hUSLFs (passages 2–6) were transfected at approximately 70% confluency using Lipofectamine 3000 (Invitrogen, Carlsbad, USA) according to the manufacturer's protocol. Overexpression plasmids of FLJ20021, XRCC6, and ADI1 were constructed, including FLAG‐tagged constructs for truncated variants of XRCC6. In parallel, siRNAs targeting FLJ20021, XRCC6, and ADI1 with the corresponding negative controls were synthesized by Syngenbio Co., Ltd. (Beijing, China).

### 2.17 5‐Ethynyl‐2′‐deoxyuridine (EdU) Assay

An EdU Imaging Kit (APExBIO, Houston, USA) was used according to the manufacturer's instructions. Briefly, cells were seeded in a 24‐well plate with EdU working solution for 2 h. Following fixation, cells were stained with click reaction solution for 30 min in the dark, followed by counterstaining with Hoechst for 15 min. Subsequently, a fluorescence microscope was captured using the ImageXpress Confocal High‐Content Imaging System (Molecular Devices, San Jose, CA, USA). EdU‐positive cells were regarded as cells with proliferative capacity. For quantitative analysis, five random fields were analyzed in three individual wells per group. The percentage of proliferating cells was calculated as (EdU‐positive nuclei/total nuclei) ×100% using MetaXpress software.

### 2.18 CCK‐8 Assay

Cell viability was assessed using the Cell Counting Kit‑8 (Beyotime). Briefly, cells were seeded in 96‑well plates and subjected to various interventions for 1–5 days. At each designated time point, 10 µL of CCK‑8 reagent was added per well, and the plates were incubated for 1 h at 37°C. Absorbance at 450 nm was then measured using an Infinite 200 PRO microplate reader (Tecan, Switzerland), with higher absorbance indicating greater cell viability.

### 2.19 Senescence‐Associated Beta‐Galactosidase (SA‐β‐Gal) Assay

Senescence detection with β‐galactosidase was according to the manufacturer's instruction of β‐galactosidase staining Kit (Beyotime). Briefly, cells were seeded into 24‐well plates at a density of 2 × 10^4^ cells per well and cultured until they reached 50%–60% confluence. Cells were fixed. Cells were treated with the staining working solution (including the X‐Gal solution and staining solutions A, B, and C) without CO_2_. Finally, quantify the percentage of blue (SA‐β‐Gal‐positive) senescent cells under a phase contrast microscope (Olympus IX51). Five random fields were analyzed in three individual wells per group. The senescence rate was calculated as (SA‐β‐Gal‐positive cells/total cells) ×100% using ImageJ‐assisted manual evaluation.

### 2.20 Flow Cytometry for Apoptosis

Apoptosis was quantified using the Annexin V‐FITC Apoptosis Detection Kit (Beyotime, Beijing, China). The procedure was conducted according to the manufacturer's instructions. In brief, cells were harvested by trypsinization (without EDTA) and washed with ice‐cold PBS. The cells were then resuspended and prepared as single‐cell suspensions. The results were analyzed on a FACSymphony A1 flow cytometer (BD Biosciences, Becton, Dickinson and Company, Franklin Lakes, USA). Data were subsequently processed using FlowJo version 10 software.

### 2.21 TUNEL Staining Assay

Terminal deoxynucleotidyl transferase dUTP nick end labeling (TUNEL) staining was performed to detect DNA fragmentation associated with apoptosis using the One‑step TUNEL FITC Apoptosis Detection Kit (APExBIO). Briefly, cells grown on coverslips were washed with PBS and fixed with 4% paraformaldehyde. After fixation, cells were permeabilized in 0.1% Triton X‑100 in PBS. The TUNEL reaction mixture was then freshly prepared according to the manufacturer's instructions and applied to the cells. After thorough washing with PBS, the nuclei were counterstained with DAPI. Fluorescence images were captured by the ImageXpress Confocal High‐Content Imaging System (Molecular Devices, San Jose, CA, USA). Five random fields were analyzed in three individual wells per group. The apoptosis index was calculated as (TUNEL‐positive nuclei/total nuclei) ×100% using MetaXpress software.

### 2.22 Establishment of Senescent Cell Models

hUSLFs at 70% confluency were treated for 2 h with 200 µM concentration of hydrogen peroxide solution. The cells were cultured at 37°C under 5% CO_2_. Subsequently, Cellular senescence was assessed by the SA‐β‐Gal assay and immunoblots of P21.

### 2.23 Animal Experiment Design

Nulliparous 7‐week‐old wild‐type ICR mice were obtained from Huafukang (Beijing, China). All of our animal experimental protocols and implementations were conducted based on the institutional guidelines of the Institutional Review Committee and authorized by the Animal Welfare and Ethics Committee of China Medical University (Shenyang, China, approval No. CMU20252259). Female mice were randomly allocated to sham, vaginal distension (VD), ovariectomized (OVX), and VD + OVX groups with 6 animals per group for different experiments. For sham, a balloon catheter was inserted intravaginally under general anesthesia without inflation; anesthesia was maintained for the same duration as VD. For VD, the balloon was inflated to 400 µL and maintained for 40 min to simulate vaginal delivery [25]. OVX was performed bilaterally to decrease estrogen levels, and the VD + OVX group underwent both OVX and VD as above.

### 2.24 In Vivo Lentivirus Infection of Tissue

All in vivo studies with lentivirus were conducted in accordance with local and national guidelines on the ethical use of animals in research as well as adhering to all regulations associated with the use of category II infectious materials. Human FLJ20021 overexpression lentiviral vectors (LV‐ FLJ20021) and control empty lentiviral vectors (LV‐vector) (Syngenbio Co., Ltd., Beijing, China) were injected into the posterior uterine wall near the cervix, and the virus titre was 1 × 10^8^TU/mL. The construct of LV‐ FLJ20021 was inserted into the pLV‐hEF1α‐mNeonGreen‐P2A‐Puro‐WPRE‐CMV‐FLJ20021 (Syngenbio Co., Ltd.) (Figure 8A). The negative control was the corresponding empty lentiviral vector. The lentiviral vector protocol was performed according to the manufacturer's instructions. In brief, we used 293T cells to confirm the transfection efficiency and determined the proper virus titre by examining the expression of FLJ20021 using RT‐qPCR. In VD+OVX mice, a midline laparotomy was performed; the posterior uterine wall near the cervix was exposed, and lentiviral suspension was slowly injected into the uterine wall adjacent to USLs. Finally, we sutured the muscle and skin layers. All procedures were conducted aseptically, and animals were monitored until recovery. Mice were sacrificed by cervical dislocation at 1 week post‐surgery. USLs were collected for RT‐qPCR (snap‐frozen) or fixed with 4% paraformaldehyde and paraffin‐embedded for histological analysis.

### 2.25 Statistical Analysis

Experiments were performed with at least three independent biological replicates. Biological replicates were defined as individual patient‐derived USL specimens, independent patient‐derived primary hUSLFs, or individual mice. All data are expressed as the mean  ±  standard deviation (SD), or non‐normally distributed continuous data as median (interquartile range). GraphPad Prism 9.1 software (GraphPad Software Inc., La Jolla, CA, USA) and ImageJ (National Institutes of Health, Bethesda, MD, USA) were used for statistical analysis. Prior to applying parametric tests, normality and homogeneity of variance were assessed. For normally distributed data, differences between two groups were analyzed using Student's two‐tailed t‐test, while differences among more than two groups were assessed using one‐way analysis of variance (ANOVA) followed by Tukey's post hoc test. For nonparametric data, the Kruskal–Wallis test and Mann–Whitney U test were applied. Categorical variables were analyzed using the chi‐square test. Statistical significance is shown as ^*^ *p* < 0.05, ^**^ *p* < 0.01, ^***^ *p* < 0.001.

## 3 Results

### 3.1 Reduced FLJ20021 in POP and Predominant Nuclear Localization

Transcriptome sequencing was performed to identify the differentially expressed lncRNAs in POP USL tissues compared with non‐POP controls, and our previous work identified FLJ20021 as a dysregulated lncRNA that may modulate ECM composition in fibroblasts [24]. RT‐qPCR analysis of USL tissues confirmed that FLJ20021 levels were significantly lower in the POP group (Figure 1A). Consistently, ISH staining demonstrated a marked reduction of FLJ20021 expression in POP specimens, with predominant nuclear localization in fibroblasts and some cytoplasmic signal (Figure 1B,C). Primary cells derived from USL tissues predominantly exhibited a spindle‐shaped morphology (Figure 1D). IF analysis of second‐passage hUSLFs showed that 96.56 ± 1.88% of cells were positive for vimentin (a fibroblast marker) and negative for Pan‐Cytokeratin and desmin (epithelial and smooth muscle markers, respectively) (Figure 1E), indicating that the majority of cultured cells were fibroblasts. RT‐qPCR further confirmed that FLJ20021 expression remained significantly lower in POP‐derived hUSLFs than in non‐POP controls (Figure 1F). Because subcellular localization of lncRNAs often informs their potential mechanisms of action, we next examined the distribution of FLJ20021. FISH and nucleocytoplasmic fractionation assays indicated that FLJ20021 was primarily localized in the nucleus (Figure 1G,H).

**FIGURE 1:**
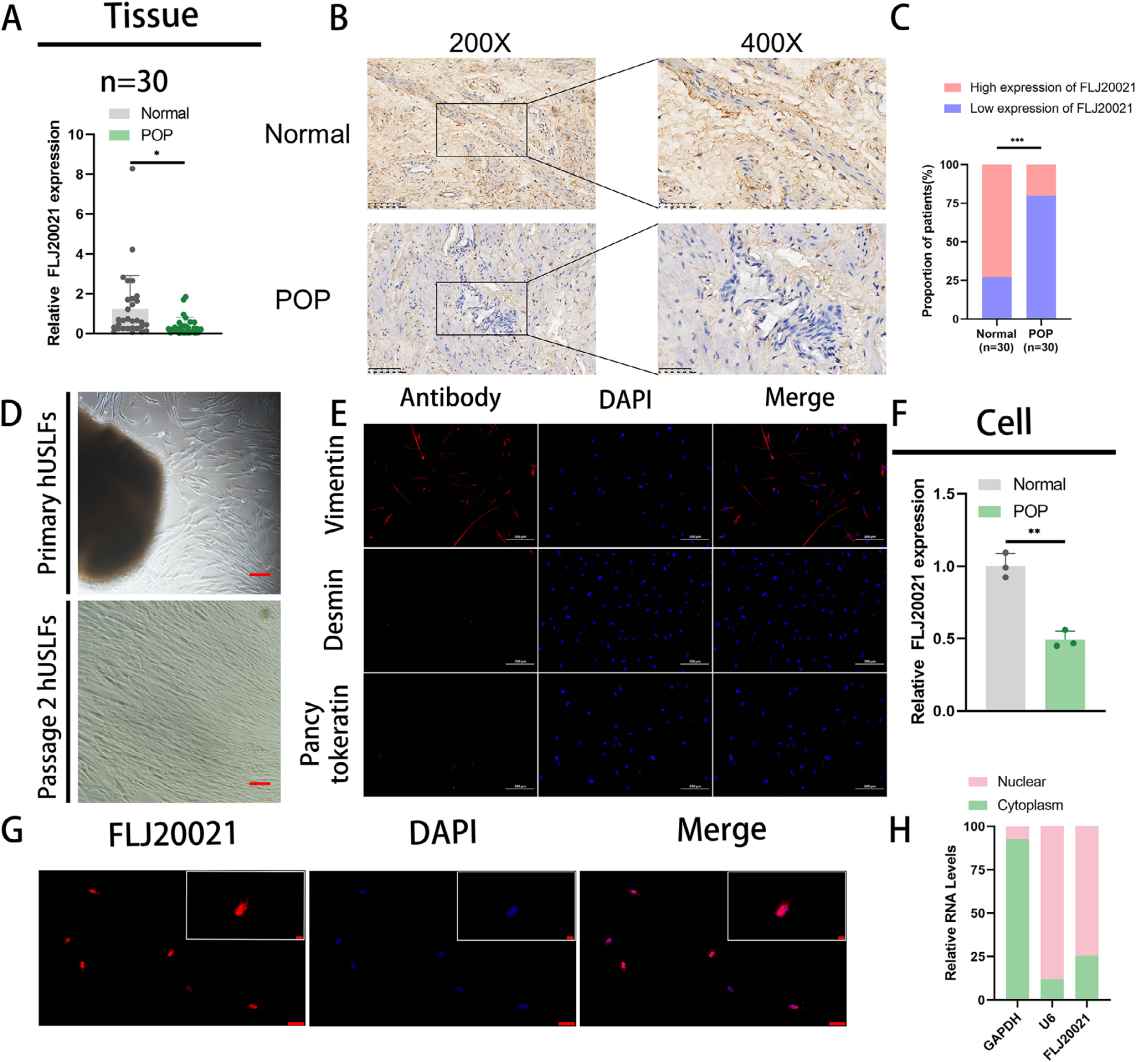
FLJ20021 is downregulated in POP and located in the nucleus. (A–C) Analysis of FLJ20021 expression in POP USL tissues (*n* = 30) and non‐POP USL tissues (*n* = 30) using RT–qPCR and ISH assays. Left scale bar = 100 µm (200×images), right scale bar = 50 µm (400×images). (D) Representative micrographs of primary and second‐passage hUSLFs. Scale bars = 100 µm. (E) IF images of second‐passage hUSLFs used to confirm fibroblast identity. Scale bars = 200 µm. (F) Expression of FLJ20021 in POP and non‐POP hUSLFs. (G) The subcellular localization of FLJ20021 was examined via FISH. Upper scale bar = 10 µm, lower scale bar = 50 µm. (H) The subcellular level of FLJ20021 in the nucleus and cytoplasm of hUSLFs was determined by nucleocytoplasmic separation and RT‐qPCR assays. Data are presented as means ± SD. ^*^ *p* < 0.05, ^**^ *p* < 0.01, ^***^ *p* < 0.001 (Student's t‐test).

### 3.2 FLJ20021 Regulates Proliferation, Apoptosis, and Senescence of hUSLFs in Vitro

To investigate the functional role of FLJ20021 in POP, three siRNAs were synthesized and transfected into POP‐derived hUSLFs. RT–qPCR confirmed that FLJ20021 expression was significantly reduced in transfected cells compared with si‐NC‐transfected controls (Figure 2A, Figure S1C). Cell viability assessed by CCK‐8 and EdU assays showed that knockdown of FLJ20021 significantly impaired hUSLFs proliferation relative to controls (Figure 2B–D, Figure S1D,E). To determine whether FLJ20021 affects apoptosis, flow cytometry and TUNEL assays were performed, demonstrating that FLJ20021 knockdown increased apoptosis (Figure 2E–H, Figure S1F,G). Moreover, SA‐β‐gal staining indicated that FLJ20021 knockdown significantly increased the proportion of senescent cells compared with controls (Figure 2I,J).

**FIGURE 2:**
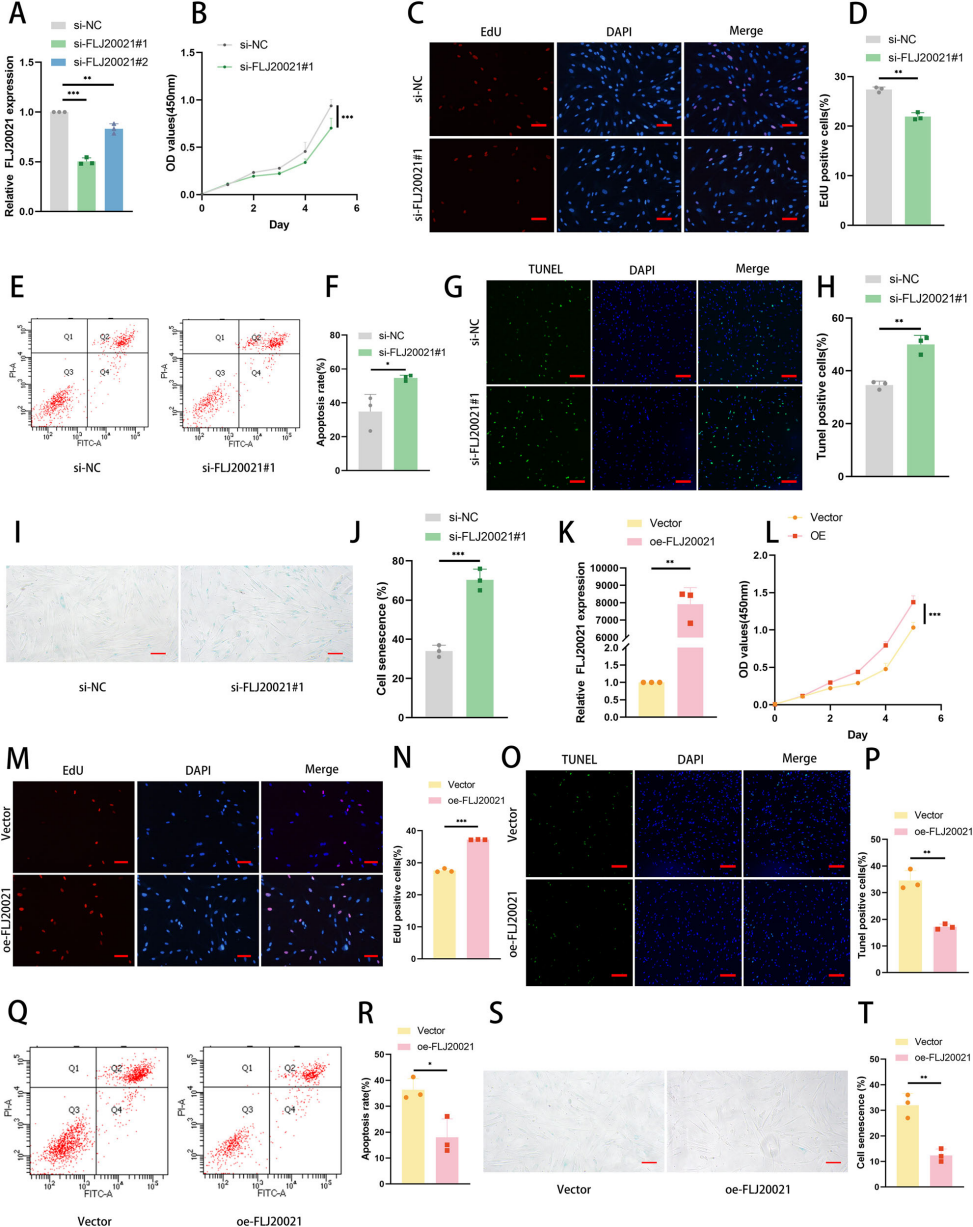
Effects of differential FLJ20021 expression levels on proliferation, apoptosis, and senescence in hUSLFs. (A) FLJ20021 was successfully silenced in hUSLFs. (B) The CCK‐8 assays confirmed that FLJ20021 depletion significantly inhibited hUSLFs growth. (C, D) The proliferation of hUSLFs transfected with si‐FLJ20021 was determined by the EdU staining assay (*n* = 3). Scale bar = 100 µm. (E, F) Flow cytometry was used to analyze apoptotic cell distribution in FLJ20021‐knockdown hUSLFs and control cells. (G, H) Apoptosis in FLJ20021‐knockdown hUSLFs was assessed by TUNEL staining (*n* = 3). Scale bar = 100 µm. These apoptosis assays demonstrate that FLJ20021 knockdown significantly increases apoptosis in hUSLFs. (I, J) The SA‐β‐gal staining assay showed that FLJ20021 knockdown obviously increased the number of senescent hUSLFs (*n* = 3). Scale bar = 100 µm. (K) The RNA level of FLJ20021 was measured by RT‐qPCR assay in hUSLFs transfected with the indicated plasmids. (L–N) Cell proliferation of hUSLFs was increased in the FLJ20021 overexpression group compared to vector controls, as assessed by the CCK‐8 and EdU assays. (O–R) Flow cytometry and TUNEL staining demonstrated that overexpression of FLJ20021 inhibited apoptosis in hUSLFs. Scale bar = 100 µm. (S, T) SA‐β‐gal staining revealed that FLJ20021 overexpression reduced SA‐β‐gal positivity (*n* = 3). Scale bar = 100 µm. Data are presented as means ± SD of three independent experiments. ^*^ *p* < 0.05, ^**^ *p* < 0.01, ^***^ *p* < 0.001 (Student's t‐test or ANOVA).

Conversely, FLJ20021 was overexpressed in POP‐derived hUSLFs via plasmid transfection, and RT‑qPCR confirmed successful overexpression (Figure 2K). CCK‐8 and EdU assays indicated that overexpression of FLJ20021 promoted the growth capability of hUSLFs (Figure 2L–N). Apoptosis assays further revealed that FLJ20021 overexpression suppressed apoptosis (Figure 2O–R). Consistently, SA‐β‐Gal staining showed that FLJ20021 overexpression significantly reduced the proportion of senescent cells relative to controls (Figure 2S,T). Collectively, these results indicate that FLJ20021 knockdown inhibits proliferation and promotes apoptosis and senescence in hUSLFs, whereas FLJ20021 overexpression exerts opposite effects.

### 3.3 FLJ20021 Interacts With RBP XRCC6 Directly in hUSLFs From POP Tissues

To gain mechanistic insight, RNA pull‐down assays coupled with MS were performed to identify FLJ20021‐interacting proteins. Agarose gel electrophoresis confirmed the successful in vitro transcription of both sense and antisense FLJ20021 RNA for subsequent RNA pull‐down assays (Figure 3A). The pulled‐down proteins were subjected to SDS‐PAGE followed by MS analysis (Figure 3B). XRCC6 was the only RBP that overlapped between the MS candidates and the online RBP Suite database (http://www.csbio.sjtu.edu.cn/bioinf/RBPsuite/) (Figure 3C). Consistently, XRCC6 was specifically pulled down by the FLJ20021 probe in hUSLFs (Figure 3D). A subsequent RIP assay further confirmed the interaction, as FLJ20021 enrichment in XRCC6 immunoprecipitates was approximately 15‐fold higher than in the IgG control (Figure 3E,F). Additionally, the fluorescence images captured by laser confocal fluorescence microscopy demonstrated that colocalization of FLJ20021 and XRCC6 occurred in both the nucleus and cytoplasm of hUSLFs (Figure 3G).

**FIGURE 3:**
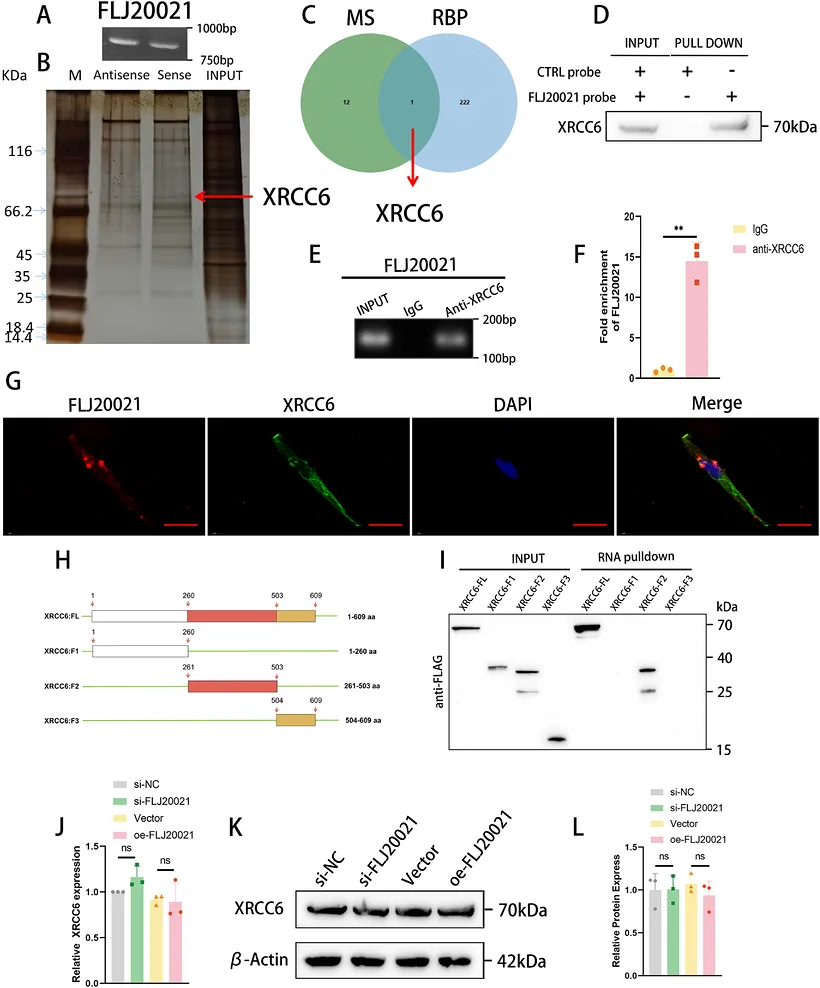
FLJ20021 physically interacts with XRCC6. (A) Agarose gel electrophoresis confirmed successful in vitro transcription of FLJ20021 RNA, displaying both sense and antisense strands. (B) The RNA pull‐down assay for identification of FLJ20021‐interacting proteins was performed. The sense and antisense strands of FLJ20021 RNA were biotinylated, refolded, and incubated with hUSLFs lysates. The red arrow represents the differential band containing the potential FLJ20021 binding proteins. (C) According to the MS and RBP Suite database analysis, the Venn diagram identified XRCC6 as the sole RBP interacting with FLJ20021. (D) Western blotting using RNA pulldown samples confirmed the binding of FLJ20021 and XRCC6. (E, F) The interaction of FLJ20021 and XRCC6 was further verified using the RIP‐qPCR assay in hUSLFs. (G) FISH+IF images demonstrated that FLJ20021 has a similar subcellular location to the XRCC6 protein in hUSLFs. Scale bar = 20 µm. (H, I) XRCC6 (609 aa) was divided into three truncations: F1 (aa 1–260), F2 (aa 261–503), and F3 (aa 504–609). Proportions were calculated as fragment length (aa)/609 × 100%. Plasmids encoding FLAG‐tagged FL or truncation mutants of XRCC6 were transfected into 293T cells and then were subjected to RNA pull‐down experiments using a biotin‐labeled FLJ20021 probe. WB analysis demonstrated that only F2 and full‐length XRCC6 fragments could interact with FLJ20021. (J–L) RT‐qPCR and WB analyses showed that XRCC6 mRNA and protein levels remained unchanged in hUSLFs following FLJ20021 silencing or overexpression. Data are presented as means ± SD of three independent experiments. ^**^ *p* < 0.01 (Student's t‐test).

To map the XRCC6 region required for binding to FLJ20021, we leveraged the domain architecture of XRCC6, which includes an N‑terminal domain, a central DNA‑binding core domain (DBD), and a C‑terminal SAP domain [26, 27]. We generated expression constructs encoding full‐length XRCC6 (XRCC6‐FL) and truncation mutants corresponding to these regions (XRCC6‐F1, 1–260 aa; XRCC6‐F2, 261–503 aa; XRCC6‐F3, 504–609 aa) (Figure 3H). Lysates from 293T cells transfected with these constructs were incubated with the biotin‐labeled FLJ20021 probe for a pull‐down assay. XRCC6‐ F2 was efficiently pulled down by FLJ20021, indicating that FLJ20021 primarily interacted with the DBD of XRCC6 (Figure 3I).

We next evaluated whether FLJ20021 regulates XRCC6 expression. Following knockdown or overexpression of FLJ20021 in hUSLFs, XRCC6 mRNA and protein levels remained unchanged (Figure 3J–L), indicating that FLJ20021 does not affect XRCC6 expression directly. Together, these results support a model in which FLJ20021 modulates XRCC6 activity through direct binding, thereby influencing downstream targets.

### 3.4 The FLJ20021‐Binding Protein XRCC6 Transcriptionally Activates ADI1 in hUSLFs

To explore downstream effectors of FLJ20021 and XRCC6 in POP pathogenesis, RNA‐seq was performed on POP‐derived hUSLFs following knockdown of FLJ20021 or XRCC6 (Figure 4A,B). Gene Ontology (GO) analysis of FLJ20021‑regulated genes revealed significant enrichment of the “protein–DNA complex” category, consistent with our finding that FLJ20021 binds the DBD of XRCC6 (Figure 4C). KEGG pathway analysis further indicated enrichment of pathways related to cellular senescence and apoptosis (Figure 4D). Notably, ADI1 was the only overlapping differentially expressed gene between the FLJ20021‐ and XRCC6‐knockdown conditions (Figure 4E), prompting us to investigate ADI1 as a candidate downstream effector of FLJ20021 and XRCC6 in POP.

**FIGURE 4:**
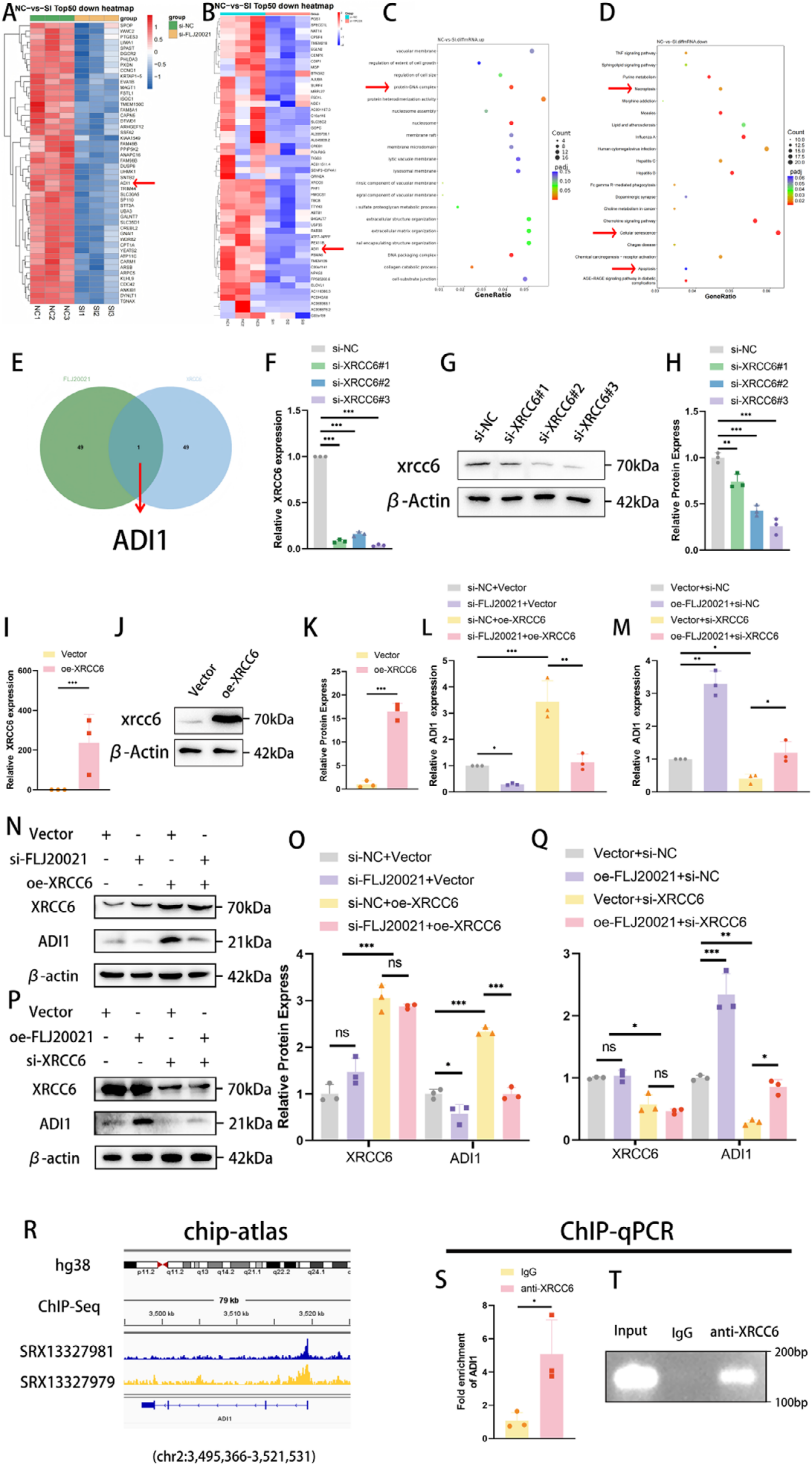
ADI1 is a downstream target regulated by FLJ20021 and XRCC6. (A, B) Heatmap of differentially expressed genes identified by RNA‐seq following knockdown of FLJ20021 or XRCC6. (C, D) GO and KEGG enrichment analyses of the differentially expressed genes after FLJ20021 knockdown. (E) A Venn diagram summarizing differentially expressed genes from RNA‐seq following FLJ20021 or XRCC6 knockdown highlighted ADI1 as the sole commonly regulated target. (F–H) The RT‐qPCR and WB assays showed that XRCC6 was successfully knocked down in hUSLFs. (I–K) The RT‐qPCR and WB assays confirmed that XRCC6 was successfully overexpressed in hUSLFs. (L–Q) The rescue RT‐qPCR and WB assays demonstrated that XRCC6 overexpression restored the reduction in ADI1 expression caused by FLJ20021 knockdown, and XRCC6 knockdown hindered the rise in ADI1 expression induced by FLJ20021 overexpression. (R) Genome browser view (ChIP‐Atlas database) illustrated XRCC6‐binding peaks in the ADI1 locus under the hg38 reference genome. (S, T) ChIP‐qPCR assays to determine the binding status of XRCC6 to the ADI1 promoter region in hUSLFs, with IgG as a negative control. Data are presented as means ± SD of three independent experiments. ^*^ *p* < 0.05, ^**^ *p* < 0.01, ^***^ *p* < 0.001 (Student's t‐test or ANOVA).

The transfection efficiency of the XRCC6 overexpression plasmid and XRCC6‐targeting siRNA in hUSLFs was confirmed by WB and RT‐qPCR (Figure 4F–K). These validated cells were then used for subsequent experiments. We then performed rescue experiments to test whether FLJ20021 regulates ADI1 through XRCC6. XRCC6 overexpression restored ADI1 mRNA and protein levels that were reduced by FLJ20021 knockdown, whereas XRCC6 silencing blunted the increase in ADI1 expression induced by FLJ20021 overexpression (Figure 4L–Q). These results indicate that FLJ20021 regulates ADI1 in an XRCC6‑dependent manner.

Although XRCC6 is best known for its essential role in DNA repair, particularly through the non‐homologous end joining (NHEJ) pathway [26, 28], emerging evidence indicates that it also associates with gene promoter regions and modulates the transcriptional activity of target genes [29, 30]. We next asked whether XRCC6 directly engages the ADI1 promoter. According to the ChIP‐Atlas database(https://chip‐atlas.org/), ChIP‐seq data for XRCC6 reveals significant peaks in the promoter‐like region of ADI1(Figure 4R). Consistent with this, our ChIP–qPCR experiments confirmed significant binding of XRCC6 to the ADI1 promoter region, with the PCR product verified by agarose gel electrophoresis (Figure 4S,T). In addition, FLJ20021 knockdown markedly decreased the binding of XRCC6 to the ADI1 promoter, whereas FLJ20021 overexpression increased it (Figure S1A,B). Collectively, these findings support that FLJ20021 modulates ADI1 expression via XRCC6, which occupies the ADI1 promoter and contributes to ADI1 transcriptional regulation in hUSLFs.

### 3.5 XRCC6 and ADI1 Mediate FLJ20021‐Dependent Proliferation, Apoptosis, and Senescence in hUSLFs

To assess the potential involvement of XRCC6 in POP, we first examined XRCC6 expression in USL tissues. XRCC6 expression was markedly decreased in USLs from POP patients compared with non‐POP controls (Figure 5A, Figure S2A,B). We next evaluated the functional impact of XRCC6 in hUSLFs. XRCC6 overexpression increased hUSLF viability, whereas XRCC6 knockdown reduced proliferation (Figure 5B,C), as verified by EdU assays. TUNEL staining further revealed that XRCC6 knockdown promoted apoptosis, whereas XRCC6 overexpression suppressed apoptosis in hUSLFs (Figure 5D,E). To test whether XRCC6 mediates the effects of FLJ20021, we performed rescue experiments. XRCC6 overexpression restored the reduced proliferation induced by FLJ20021 knockdown, whereas XRCC6 depletion abrogated the proliferation‑enhancing effect of FLJ20021 overexpression, as shown by EdU assays (Figure 5F–I). Consistently, TUNEL assays revealed that the elevated apoptosis rate in FLJ20021‐silenced hUSLFs was mitigated by XRCC6 overexpression, whereas XRCC6 knockdown counteracted the anti‐apoptosis effect of FLJ20021 overexpression (Figure 5J–M).

**FIGURE 5:**
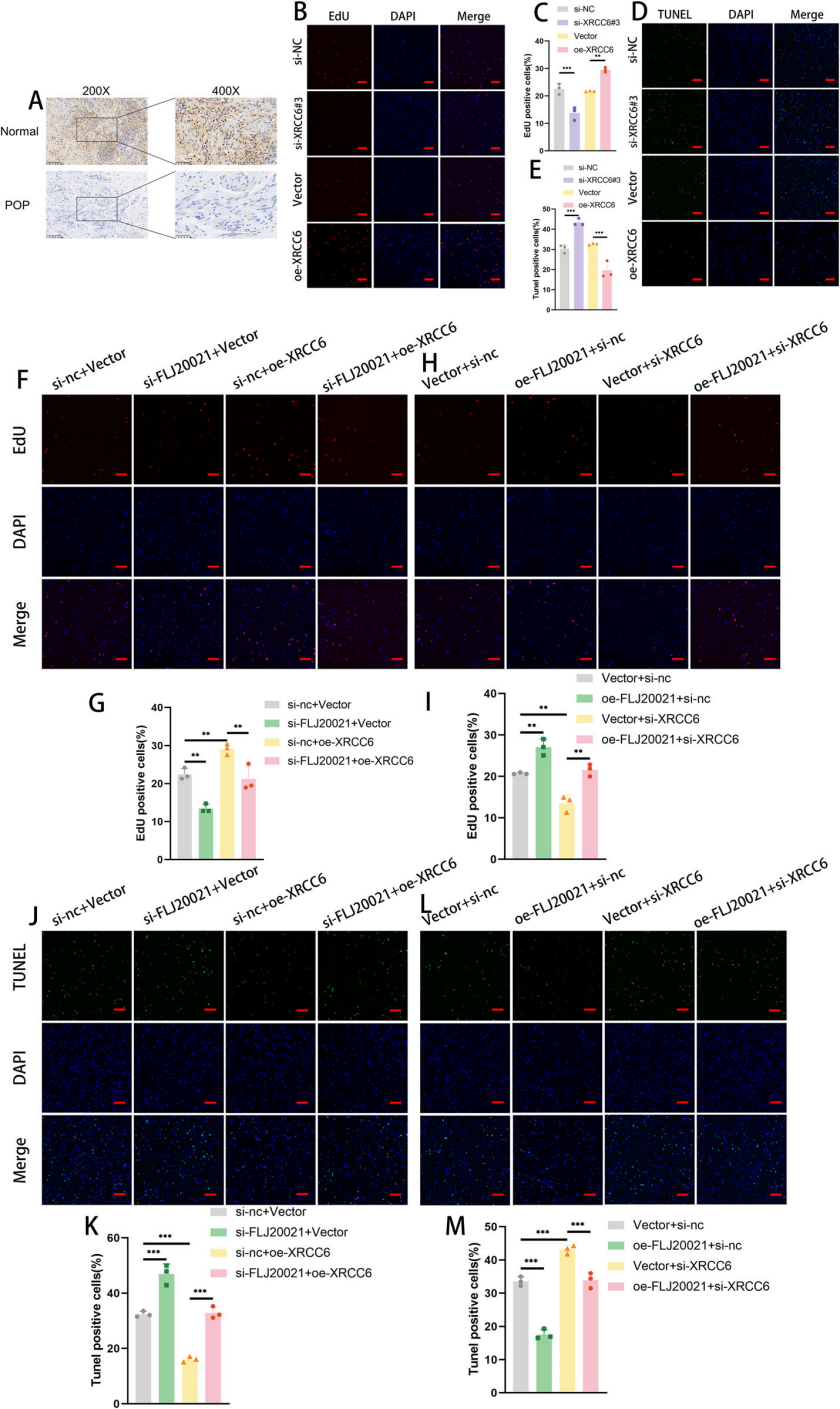
Regulation of XRCC6 expression affects hUSLFs proliferation and apoptosis. (A) The expression levels of XRCC6 proteins in POP (*n* = 20) and non‐POP (*n* = 20) USL tissues were determined by IHC analysis. Left scale bar = 100 µm (200×images), right scale bar = 50 µm (400×images). (B, C) The proliferation of hUSLFs transfected with XRCC6‐specific siRNAs or XRCC6 plasmids was determined by EdU staining. XRCC6 knockdown reduced the percentage of EdU‐positive hUSLFs, whereas XRCC6 overexpression increased it. (D, E) TUNEL staining detected an increased apoptosis rate with XRCC6 knockdown and a decreased apoptosis rate with XRCC6 overexpression. (F–I) The rescue EdU assay showed that the inhibitory effect of FLJ20021 knockdown on hUSLF growth was rescued by XRCC6 overexpression, and the promotive effect of FLJ20021 overexpression on cell growth was reversed by XRCC6 knockdown in hUSLFs. (J–M) The rescue TUNEL staining showed that the promotive effect of FLJ20021 knockdown on hUSLF apoptosis was rescued by XRCC6 overexpression, and the inhibitory effect of FLJ20021 overexpression on apoptosis was reversed by XRCC6 knockdown in hUSLFs (*n* = 3). Scale bar = 100 µm. Data are presented as means ± SD of three independent experiments. ^*^ *p* < 0.05, ^**^ *p* < 0.01, ^***^ *p* < 0.001 (Student's t‐test or ANOVA).

Patients with POP exhibited significantly lower ADI1 expression compared with controls (Figure 6A, Figure S2C,D). To define the role of ADI1 in FLJ20021‐mediated phenotypes in POP‐derived hUSLFs, we performed rescue experiments. An ADI1 expression plasmid and an ADI1‐targeting siRNA were transfected into POP‐derived hUSLFs. The efficiency of ADI1 overexpression and knockdown was confirmed by WB and RT‐qPCR. ADI1 mRNA and protein levels were significantly increased in the overexpression group and decreased in the knockdown group relative to controls (Figure 6B–G). Functionally, EdU assays demonstrated that ADI1 overexpression rescued the proliferation impairment caused by FLJ20021 knockdown in hUSLFs, whereas ADI1 depletion abrogated the proliferation‐promoting effect of FLJ20021 overexpression (Figure 6H–K). TUNEL assays further revealed that ADI1 overexpression mitigated the pro‐apoptotic effect of si‐FLJ20021, while ADI1 knockdown counteracted the apoptosis‐suppressing impact of FLJ20021 overexpression (Figure 6L–O). Consistently, SA‐β‐gal staining showed that overexpression of ADI1 decreased the proportion of SA‑β‑Gal‑positive cells and alleviated the increase in cellular senescence induced by FLJ20021 knockdown (Figure 6P,Q), supporting a protective role of ADI1 against senescence in hUSLFs.

**FIGURE 6:**
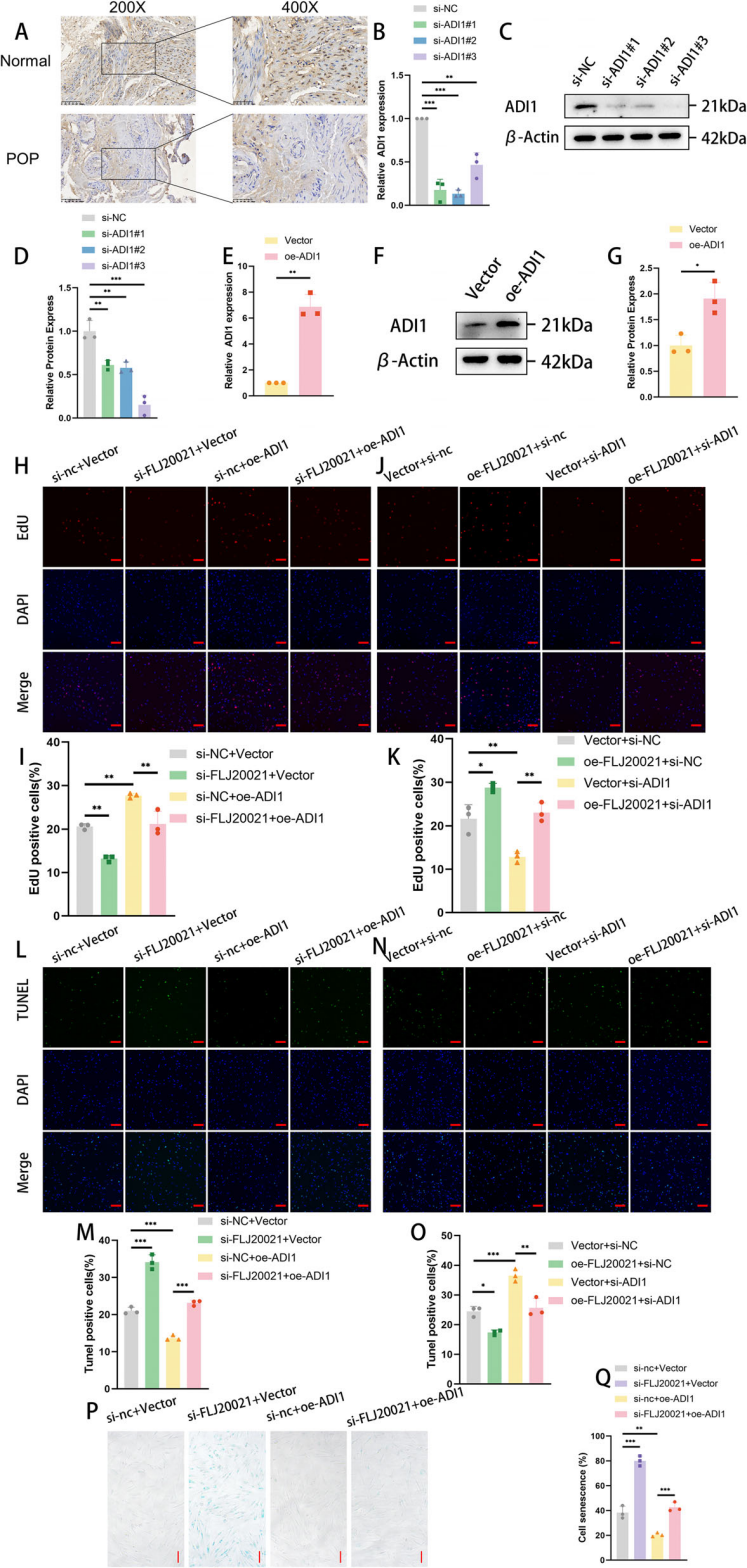
ADI1 was down‐regulated and rescued FLJ20021‐induced hUSLFs proliferation, apoptosis, and senescence. (A)Representative images of IHC results showed that POP USL tissues (*n* = 20) had lower ADI1 abundance when compared with non‐POP USL tissues (*n* = 20). Left scale bar = 100 µm (200×images), right scale bar = 50 µm (400×images). (B–G) RT‐qPCR and WB analyses confirmed that ADI1 siRNA and plasmid transfections were effective in hUSLFs. (H–K) Rescue EdU assays assessed hUSLFs proliferation following ADI1 overexpression in si‐FLJ20021 hUSLFs and ADI1 knockdown in oe‐FLJ20021 hUSLFs (*n* = 3). Scale bar = 100 µm. (L–O) Rescue of hUSLFs apoptosis by ADI1 overexpression in si‐FLJ20021 hUSLFs and by ADI1 knockdown in oe‐FLJ20021 hUSLFs, as determined by TUNEL staining (*n* = 3). Scale bar = 100 µm. (P, Q) Rescue impact on SA‐β‐Gal staining by ADI1 overexpression in si‐FLJ20021 hUSLFs (*n* = 3). Scale bar = 100 µm. Data are presented as means ± SD of three independent experiments. ^*^ *p* < 0.05, ^**^ *p* < 0.01, ^***^ *p* < 0.001 (Student's t‐test or ANOVA).

### 3.6 FLJ20021 Contributes to ECM Remodeling via XRCC6–ADI1 Pathway in hUSLFs

In accordance with prior studies, cellular senescence has been implicated in POP [31], and POP fibroblasts exhibit altered ECM metabolism [32]. In USLs, IHC assays showed significantly reduced COLI and increased P21 in POP compared with controls (Figure 7A,B). To investigate whether senescence was accompanied by ECM imbalance in hUSLFs, we established an oxidative stress‐induced senescence model using H_2_O_2_ and assessed senescence by SA‐β‐gal staining and P21 protein expression. As shown in Figure 7C,D and Figure S1H,I, H_2_O_2_ increased SA‑β‑Gal activity and P21 protein expression, confirming senescence induction. WB further showed decreased COL I, COL III, TIMP1, and TIMP2 levels, together with increased MMP2 and MMP9 levels in the H_2_O_2_ group (Figure 7E,F), indicating disrupted ECM balance. These findings support an association between cellular senescence and ECM remodeling‐related changes in hUSLFs.

**FIGURE 7:**
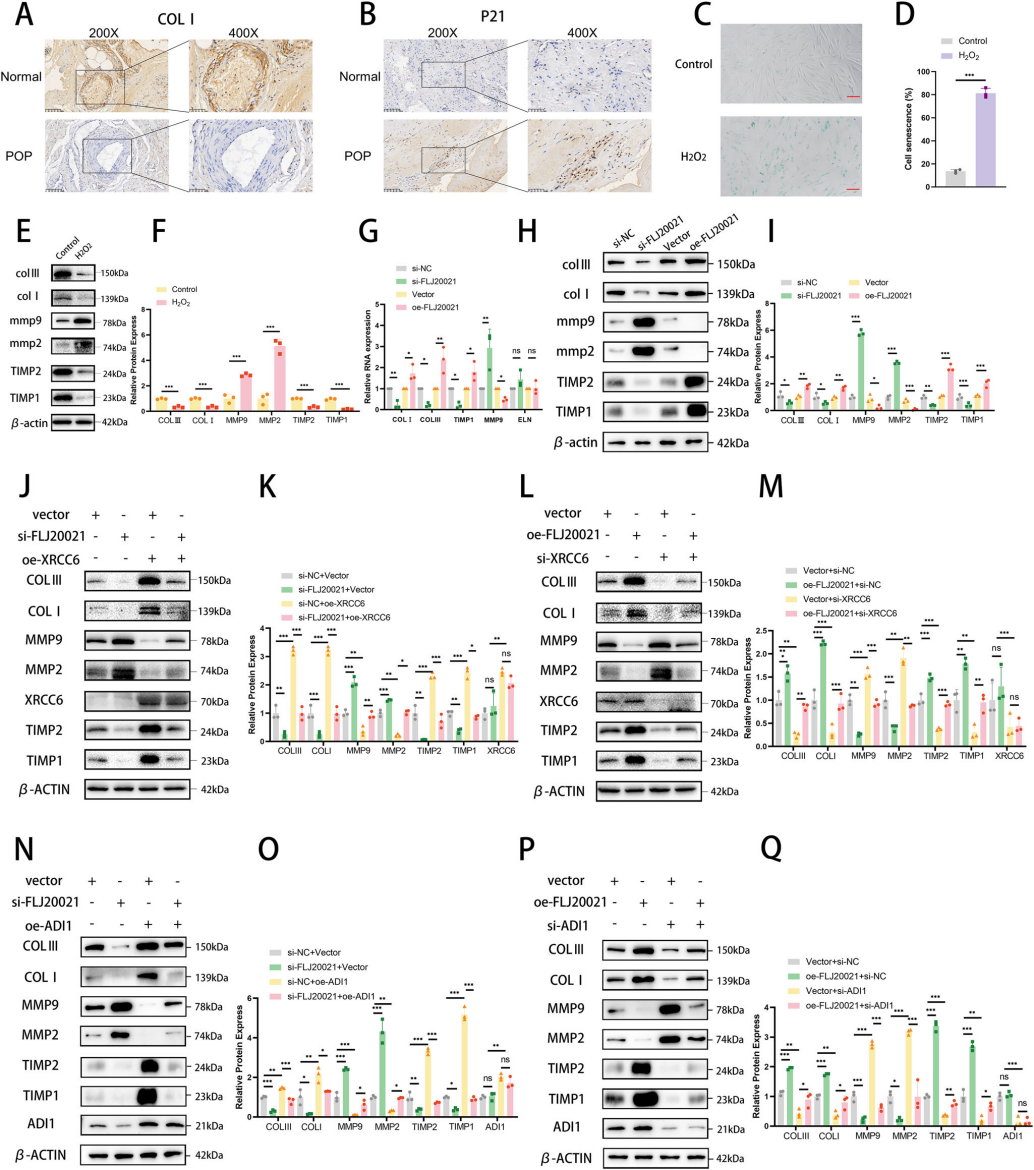
FLJ20021 regulates ECM components in hUSLFs via the XRCC6–ADI1 axis. (A, B) Representative IHC of USL tissues from non‐POP (*n* = 20) and POP patients (*n* = 20) stained for COL I and P21. Left scale bar = 100 µm (200×images), right scale bar = 50 µm (400×images). (C, D) SA‐β‐Gal staining showed senescent hUSLFs after H_2_O_2_ treatment, with proportions quantified using ImageJ (*n* = 3). Scale bar = 100 µm. (E, F) COL I, COL III, TIMP1, TIMP2, MMP2, and MMP9 expression in H_2_O_2_‐treated hUSLFs by WB. (G) RT‐qPCR evaluated the effect of FLJ20021 on the mRNA levels of ECM factors in hUSLFs. (H, I) WB analysis of ECM proteins in hUSLFs following FLJ20021 overexpression or knockdown. (J–M) WB analysis revealed that in hUSLFs, XRCC6 overexpression attenuated ECM degradation induced by knockdown of FLJ20021, whereas XRCC6 knockdown reversed ECM stabilization caused by overexpression of FLJ20021. (N, O) WB analysis of ADI1 and ECM proteins in hUSLFs revealed that the upregulation of ECM degradation markers (MMP9 and MMP2) and downregulation of ECM stabilization proteins (COL I, COL III, TIMP1, and TIMP2) induced by FLJ20021 knockdown was rectified by ADI1 overexpression. (P, Q) WB demonstrated that ADI1 knockdown counteracted the increase in proteins that stabilize the ECM and the decrease in proteins that degrade the ECM caused by FLJ20021 overexpression. Data are presented as means ± SD of three independent experiments. ^*^ *p* < 0.05, ^**^ *p* < 0.01, ^***^ *p* < 0.001 (Student's t‐test or ANOVA).

We next investigated whether FLJ20021 regulates ECM‐related components in hUSLFs. RT‐qPCR was used to measure the expression levels of COL III, COL I, MMP9, TIMP1, and Elastin (ELN) in hUSLFs under conditions of varying FLJ20021 expression. The results showed that FLJ20021 overexpression upregulated COLIII, COLI, and TIMP1 while downregulating MMP9. Conversely, FLJ20021 knockdown led to an increase in MMP9 levels, accompanied by a reduction in COLIII, COLI, and TIMP1 expression. Notably, ELN expression remained unchanged under both conditions (Figure 7G). Consistently, WB analysis confirmed that FLJ20021 overexpression increased COLI, COLIII, TIMP1, and TIMP2 levels while decreasing MMP9 and MMP2 levels, whereas FLJ20021 knockdown enhanced MMP9 and MMP2 and reduced levels of COL I, COL III, TIMP1, and TIMP2 (Figure 7H,I).

To determine whether XRCC6 mediates these effects, we performed rescue experiments. XRCC6 overexpression attenuated the ECM‐degrading marker profile induced by FLJ20021 knockdown (Figure 7J,K), whereas XRCC6 knockdown attenuated the increase in COL I, COL III, TIMP1, and TIMP2 and the decrease in MMP9 and MMP2 caused by FLJ20021 overexpression in hUSLFs (Figure 7L,M). Because ADI1 functions downstream of FLJ20021 in an XRCC6‐dependent manner, we further examined whether ADI1 is required for ECM regulation. ADI1 interference effectively reversed the ECM‐related alterations induced by FLJ20021, as verified by WB assays (Figure 7N–Q). Moreover, qPCR analysis showed that ADI1 overexpression increased, whereas ADI1 knockdown reduced, NRF2 and SOD1 mRNA expression (Figure S1J,K), suggesting a potential role for ADI1 in antioxidant regulation. In summary, these results support that FLJ20021 maintains an ECM‐preserving marker profile and limits apoptosis and senescence‐associated changes in hUSLFs through an XRCC6‐dependent ADI1 pathway.

### 3.7 FLJ20021 Attenuates OVX‐ and VD‐Induced ECM Dysregulation in Vivo

Consistent with prior reports that mechanical trauma and estrogen deficiency influence POP‐associated ECM remodeling [33, 34], we established in vivo mouse models of VD, OVX, and combined VD+OVX (Figure 8B). None of the OVX, VD, or VD+OVX regimens in this cohort produced overt uterine or vaginal wall prolapse within the observation window. IHC staining of USL‑analogous supportive tissues showed significantly decreased COL I and increased MMP9 in the VD or OVX groups compared with the Sham group (Figure 8C–E). Consistently, Masson's trichrome staining revealed reduced collagen content and a looser, more disorganized collagen architecture in the VD and OVX groups, with the most pronounced alterations observed in the VD+OVX group (Figure S2E). Notably, the combined VD+OVX model produced a more pronounced effect on ECM dysregulation than either insult alone, with reduced COL I, COL III, TIMP1, and TIMP2 and increased MMP2 and MMP9. Concordantly, WB showed decreased XRCC6 and ADI1 expression (Figure 8G,H). These findings indicate that ovarian hormone deprivation and parturition‐related mechanical trauma converge to exacerbate ECM dysregulation in the USLs and coincide with reduced XRCC6 and ADI1 expression.

**FIGURE 8:**
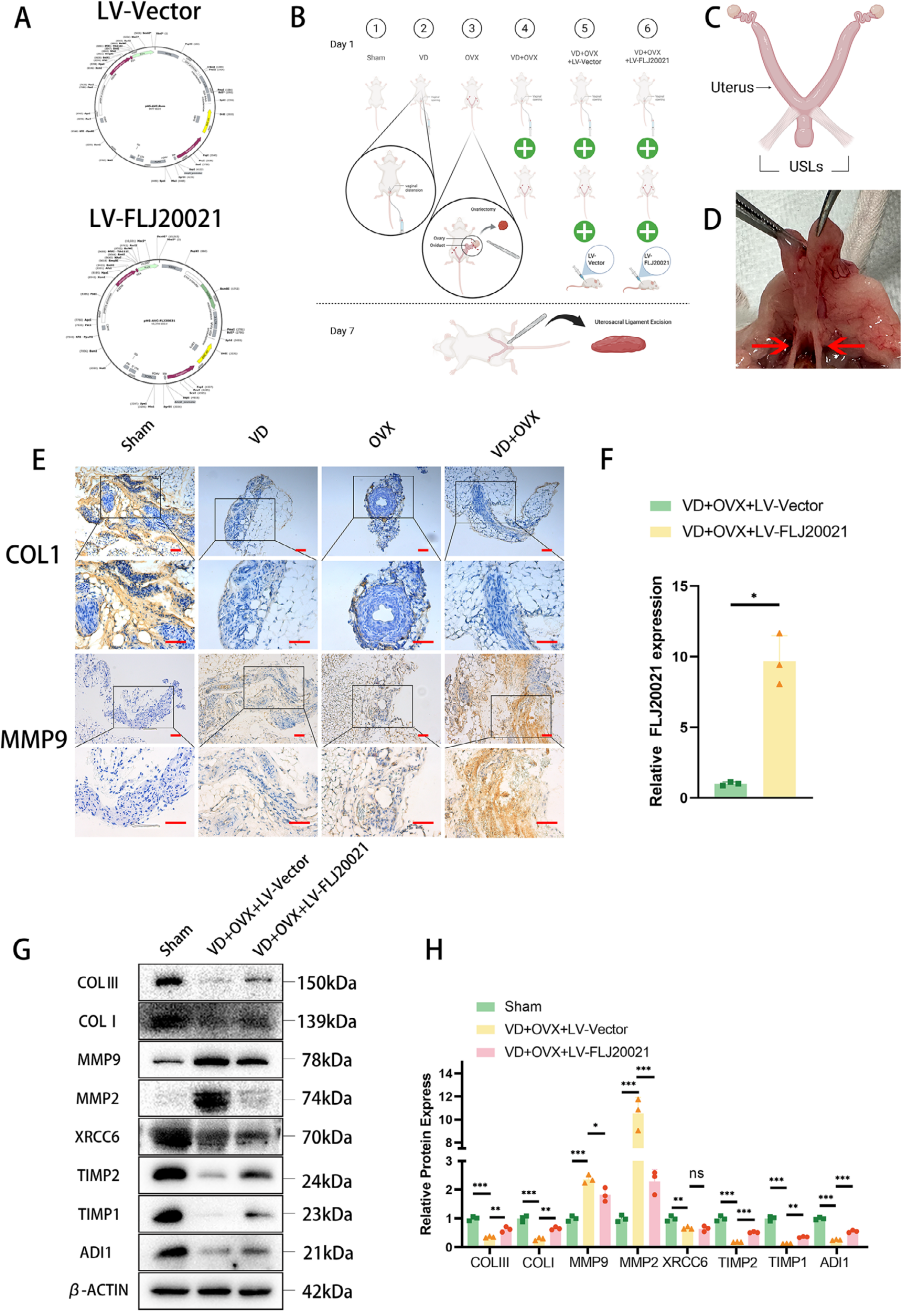
In vivo evaluation of FLJ20021 in POP‐relevant VD/OVX injury models. (A) The map of LV‐vector and LV‐FLJ20021. (B) Schematic of POP‐relevant VD/OVX injury models and overexpression of FLJ20021 intervention. (C, D) Diagram demonstrating the anatomy of murine pelvic support and the location of the USLs used for tissue collection. (E) Representative USL IHC staining of COL I and MMP9 in Sham, VD, OVX, and VD+OVX mice. Scale bar = 50 µm. (F) FLJ20021 mRNA levels in mouse USLs measured by RT‐qPCR. (G, H) WB to analyze the expression of ECM metabolism‐related proteins, XRCC6, and ADI1 in the VD+OVX injury model after FLJ20021 overexpression. Overexpression of FLJ20021 reversed the VD+OVX injury‐induced downregulation of COL I, COL III, TIMP1, and TIMP2 and the upregulation of MMP9 and MMP2, and restored ADI1 relative to LV‐Vector. Data are presented as means ± SD of three independent experiments. ^*^ *p* < 0.05, ^**^ *p* < 0.01, ^***^ *p* < 0.001 (Student's t‐test or ANOVA).

To investigate whether FLJ20021 modulates ECM remodeling in vivo, we delivered an FLJ20021‐expressing lentiviral vector in the combined VD+OVX model. RT‐qPCR analyses confirmed efficient FLJ20021 overexpression following lentiviral delivery (Figure 8A,F). FLJ20021 overexpression attenuated ECM dysregulation—restoring COL I, COL III, TIMP1, and TIMP2 and reducing MMP2 and MMP9—and partially restored ADI1 expression toward sham levels (Figure 8G,H). Collectively, these in vivo data are consistent with the FLJ20021–XRCC6–ADI1 regulatory model.

Together, these results support that FLJ20021 and XRCC6 functionally interact and converge on ADI1 to modulate ECM homeostasis, the MMP–TIMP balance, and cellular senescence and apoptosis in hUSLFs, as depicted in the graphical abstract.

## 4 Discussion

POP is a common degenerative gynecologic disorder, particularly in postmenopausal women [35], and is characterized by the downward displacement of pelvic organs such as the uterus, bladder, or rectum, significantly impairing quality of life [36]. Despite its high prevalence, the precise molecular mechanisms underlying POP remain incompletely defined. Traditional concepts emphasize anatomic and mechanical failure of pelvic floor support [37, 38]; however, emerging research indicates that biochemical changes in pelvic supportive tissues, such as alterations in COL I, COL III, and MMPs, also contribute to POP development [39]. Fibroblasts are central to pelvic‐tissue integrity because they synthesize and remodel major ECM components that determine tensile strength and elasticity. Consistent alterations in ECM composition, including reduced structural proteins and increased matrix‐degrading enzymes, have been reported in POP [40]. Importantly, fibroblast senescence has been implicated in POP‐associated matrix catabolism [1]. In line with this concept, our H_2_O_2_‐induced senescence model in primary hUSLFs exhibited increased MMP2/MMP9 with decreased COL I/COL III and TIMP1/TIMP2, supporting a senescence‐associated ECM‐degradative phenotype.

Recent studies have highlighted non‐coding RNAs as regulators of fibroblast fate and ECM remodeling relevant to POP [41, 42, 43]. Consistent with reports from our group and others, FLJ20021 is predominantly nuclear [44]. Nuclear lncRNAs frequently act as versatile molecular scaffolds or guides, recruiting and coordinating the activity of specific RBPs to fine‐tune gene expression, and these interactions are critical for modulating both transcriptional and post‐transcriptional processes. A classic example is HOTAIR, which binds Polycomb Repressive Complex 2 (PRC2) to promote trimethylation of histone H3 lysine 27 (H3K27me3) deposition and repress transcription, as first described by Rinn et al. [45] and subsequently reinforced by Gupta et al. [46]. Extending this paradigm to POP‐relevant fibroblasts, we provide mechanistic evidence that FLJ20021 directly interacts with XRCC6 in the nucleus, where XRCC6 binds to and promotes ADI1 transcription. This activation is associated with decreased cellular apoptosis and attenuated senescence, which is accompanied by ECM stabilization, including increased COL I, COL III, TIMP1, and TIMP2 and decreased MMP9 and MMP2. Notably, ELN expression remained unchanged upon FLJ20021 modulation, which may reflect distinct mechanisms governing collagen vs. elastin remodeling during ECM turnover. Collagen (COL I and COL III) is the primary structural component of the ECM, with their triple‐helical structure conferring significant mechanical strength to tissues. Despite this inherent stability, specific MMPs can recognize and degrade collagen during ECM remodeling, thereby facilitating tissue restructuring. In contrast, ELN degradation relies on the interaction of MMPs with their exosites, which is fundamentally distinct from the direct binding and localized unwinding required for collagen degradation [47]. These findings suggest that the regulatory mechanism of FLJ20021 primarily influences the MMP/TIMP system associated with dynamic collagen remodeling, while not substantially activating the pathways responsible for ELN degradation, thereby maintaining stable ELN expression.

XRCC6 encodes Ku70, which forms a stable heterodimer with Ku80 to mediate classical non‐homologous end joining (NHEJ) by binding DNA double‐strand break (DSB) ends and recruiting DNA‐dependent protein kinase catalytic subunit (DNA‐PKcs) [48, 49, 50]. XRCC6 deficiency contributes to genomic instability during replicative aging by altering DSB repair dynamics. As aging progresses, diminished XRCC6 recruitment at break sites compromises NHEJ, forcing cells to rely on more error‐prone repair pathways that generate deletions and microhomologies, thereby highlighting a dynamic relationship between XRCC6 function and replicative aging [51]. Beyond DNA repair, XRCC6 has also been implicated in fibroblast biology. For example, XRCC6 interacts with FLIP, and the stability of this complex is regulated by SIRT1‐mediated deacetylation, which can protect fibroblasts from apoptosis [52]. Notably, the DBD of XRCC6 can contribute to promoter regulation; for instance, XRCC6 cooperates with SIX1 to form a Ku70–SIX1 complex that binds the GPT2 promoter and enhances GPT2 transcription, thereby supporting metabolic programs that promote cancer cell growth and migration [29].

A notable observation in our study is that altering FLJ20021 expression did not significantly change XRCC6 mRNA or protein abundance. This suggests that FLJ20021 might serve as a molecular scaffold to interact with XRCC6 to form a functional complex important for regulating ADI1 expression. We employed RNA pull‐down and RIP assays to demonstrate that FLJ20021 directly interacts with XRCC6, and domain mapping indicates that the DBD of XRCC6 is essential for forming the FLJ20021‐XRCC6 complex. Functionally, overexpression of XRCC6 effectively reversed ADI1 expression and mitigated apoptosis and ECM degradation induced by FLJ20021 knockdown, whereas XRCC6 depletion attenuated the protective effects of FLJ20021 overexpression. ChIP‐qPCR further demonstrated XRCC6 occupancy at the ADI1 promoter, supporting a model in which the FLJ20021–XRCC6 complex promotes ADI1 transcription. Moreover, ADI1 interference effectively reversed the FLJ20021‐induced alterations in ECM‐related markers, senescence, and cell viability. Collectively, these results support that FLJ20021 acts as a molecular scaffold to facilitate XRCC6‐mediated ADI1 transcription, thereby regulating hUSLF proliferation, aging‑related phenotypes, and ECM remodeling.

RNA sequencing and rescue experiments identified ADI1 as a functional downstream effector of the FLJ20021–XRCC6 axis. Although the precise biochemical events downstream of ADI1 were not directly examined in the present study, several preliminary hypotheses and possible directions may plausibly explain how ADI1 modulates fibroblast senescence and ECM remodeling in POP‐relevant hUSLFs. First, ADI1 is a key enzyme in the methionine salvage pathway, also known as the 5′‐methylthioadenosine (MTA) cycle, and contributes to methionine recycling and intracellular S‐adenosylmethionine (SAM) homeostasis [53]. Reduced ADI1 expression may therefore influence methylation‐dependent gene regulation, as SAM is the principal methyl donor for DNA and histone methylation, thereby favoring a catabolic transcriptional program involving senescence‐related and matrix‐remodeling genes [54]. Second, disruption of methionine salvage may impair methionine‐associated one‐carbon metabolism and disturb cellular redox homeostasis. This possibility is biologically plausible because one‐carbon metabolism supports both NADPH generation and transsulfuration‐linked glutathione production, thereby contributing to antioxidant defense [55, 56, 57]. In the context of our findings, ADI1 perturbation was associated with altered expression of oxidative stress‐related markers, and oxidative stress‐induced senescence reproduced the ECM‐degrading phenotype in hUSLFs. Third, ADI1 has been reported to interact with the cytoplasmic tail of MT1‑MMP (MMP14) and suppress MT1‑MMP‐dependent processes [58]. Because MT1‑MMP can initiate a proteolytic cascade that activates gelatinases (MMP2 and MMP9) and promotes collagen/gelatin turnover [59, 60], an ADI1–MT1‑MMP axis may offer a plausible mechanism connecting ADI1 to ECM remodeling in hUSLFs. Importantly, these mechanisms may act in parallel. Thus, our data support ADI1 as a plausible and functionally important downstream mediator of the FLJ20021–XRCC6 pathway, while the relative contributions of redox regulation, SAM‐dependent epigenetic control, and MMP‐axis modulation require direct testing in future studies.

We sought supportive in vivo evidence using POP‐relevant murine injury paradigms that incorporate vaginal distension and ovariectomy, two established risk‐factor surrogates capturing mechanical trauma and hormonal deprivation. Across these models, XRCC6 and ADI1 were coordinately reduced in USL‐analogous tissues alongside ECM‐marker imbalance, and FLJ20021 overexpression partially restored ADI1 and ECM‐related readouts in vivo. Although these models did not produce overt prolapse during the observation window, the concordant molecular changes provide in vivo consistency with the in vitro mechanism and suggest that risk‐factor‐associated tissue stress may converge on the FLJ20021–XRCC6–ADI1 pathway. Notably, ECM remodeling may occur even in the absence of clinically overt prolapse.

Several limitations should be considered. First, these murine paradigms represent risk‐factor rather than anatomical prolapse models, and species differences and model constraints limit direct extrapolation to human POP. Second, our work is centered on hUSLFs and does not capture multicellular interactions across the pelvic floor (e.g., fibroblast–epithelial or fibroblast–smooth muscle crosstalk), so paracrine signaling and mechanical microenvironment effects were not directly tested. Third, the lentiviral construct used in the animal experiments expressed human FLJ20021, and a curated endogenous murine ortholog of FLJ20021 has not been established. Therefore, the in vivo findings should be interpreted as a heterologous gain‐of‐function experiment demonstrating that ectopic human FLJ20021 can modulate ADI1 and ECM‐related readouts in the mouse tissue environment. Finally, the single‐center clinical cohort and modest sample size limited covariate‐adjusted and subgroup analyses, and clinical associations should be validated in larger cohorts.

## 5 Conclusion

Our study identifies FLJ20021 as a POP dysregulated lncRNA and demonstrates in primary hUSLFs that FLJ20021 regulates proliferation, apoptosis, senescence, and ECM composition. Mechanistically, FLJ20021 binds XRCC6, facilitates XRCC6 occupancy at the ADI1 promoter, and promotes XRCC6‐dependent ADI1 transcription. Consistent with these in vitro findings, POP‐relevant murine injury models show coordinated reduction of XRCC6 and ADI1 accompanied by ECM imbalance, and FLJ20021 overexpression restores ADI1 and ECM‐related readouts in vivo. Although the upstream regulation of this pathway and additional downstream effectors remain to be defined, our work provides a mechanistic framework linking the FLJ20021‐XRCC6‐ADI1 pathway to hUSLFs fate and ECM remodeling relevant to POP, and supports this pathway as a focus for future mechanistic and translational studies.

## Author Contributions

**YanFeng Yang**: conceptualization, investigation, Writing – original draft, funding acquisition, methodology, validation, Writing – review and editing, software, supervision. **BingJie Rui**: conceptualization, investigation, methodology, data curation, resources, formal analysis, visualization, writing – review and editing, writing – original draft. **GuangHai Rui**: validation, project administration. **YiNan Duan**: validation, resources. **Ji Hyang Kim**: writing – review and editing, project administration.

## Funding

This work was supported by the Fushun Social Science Planning Project, China (grant number: fskghktlx‐2024‐009), and by a grant from the Korea Health Technology R&D Project through the Korea Health Industry Development Institute (KHIDI), funded by the Ministry of Health and Welfare, Republic of Korea (Grant number: RS‐2025‐02213795).

## Ethics Declaration

The acquisition and use of patient tissues were permitted based on the acquisition of informed consent according to the protocol approved by the Institutional Ethics Committee of Shengjing Hospital (approval number: 2024PS020K). This study was conducted in accordance with the principles of the Declaration of Helsinki. All animal procedures were approved by the China Medical University Animal Welfare and Ethics Committee (approval number: CMU20252259) and were conducted at the university's Animal Center. The study involving animals was conducted following the Basel Declaration.

## Conflicts of Interest

The authors declare no conflicts of interest.

## Supporting information




**Supporting File 1**: advs78346‐sup‐0001‐SuppMat.docx.

**Supporting File 2**: advs78346‐sup‐0002‐TableS1.docx.

## Data Availability

The data that support the findings of this study are openly available in Zenodo at: doi.org/10.5281/zenodo.18835796 and doi.org/10.5281/zenodo.18844497.
