# Supplementary material for: LncRNA FLJ20021 Restrains Fibroblast Senescence and Matrix Remodeling in Pelvic Organ Prolapse by Promoting XRCC6‐Dependent ADI1 Transcription
Source: Adv Sci (Weinh). 2026 Oct 25:e78346. Online ahead of print. doi: 10.1002/advs.78346 (PMC13696884; doi:10.1002/advs.78346)
Supplement: Supplementary file 1 [file ADVS-9999-e78346-s001.docx]

**Fig S1. (A, B)** ChIP-qPCR was used to detect the binding of XRCC6 to the ADI1 promoter in hUSLFs after FLJ20021 knockdown or overexpression. **(C)** RT–qPCR analysis of the knockdown efficiency of si-FLJ20021#3 in hUSLFs. **(D, E)** EdU assay in hUSLFs after FLJ20021 knockdown. (n=3). Scale bar=100μm. **(F, G)** TUNEL assay in hUSLFs after FLJ20021 knockdown. (n=3). Scale bar=100μm. **(H, I)** WB analysis of P21 expression in hUSLFs after H₂O₂ treatment. **(J, K)** RT–qPCR analysis of NRF2 and SOD1 mRNA expression in hUSLFs after ADI1 knockdown or overexpression. Data are presented as means±SD of three independent experiments. *p < 0.05, **p < 0.01, ***p < 0.001 (Student’s t-test).

Fig S2. **(A-D)** WB illustrating the XRCC6 and ADI1 expression in POP USL tissues and non-POP USL tissues. **(E)** Representative Masson’s trichrome staining of USL tissues from mice. Scale bar = 20 µm. Data are presented as means±SD. *p < 0.05 (Student’s t-test).
