# Supplementary material for: LncRNA FLJ20021 Restrains Fibroblast Senescence and Matrix Remodeling in Pelvic Organ Prolapse by Promoting XRCC6‐Dependent ADI1 Transcription
Source: Adv Sci (Weinh). 2026 Oct 25:e78346. Online ahead of print. doi: 10.1002/advs.78346 (PMC13696884; doi:10.1002/advs.78346)
Supplement: Supplementary file 2 [file ADVS-9999-e78346-s002.docx]

Table S1. Baseline characteristics of the clinical cohort

| **Characteristic** | **POP (n=30)** | **Non-POP (n=30)** | **P value** |
| --- | --- | --- | --- |
| Age (years) | 55.5±7.8 | 56.0±10.7 | 0.837 |
| BMI (kg/m²) | 27.5 (25.0, 31.2) | 26.0 (24.7, 31.0) | 0.543 |
| Parity | 1 (1, 1) | 1 (1, 1) | 0.504 |
| Years postmenopause (years) | 6.0 (1.3, 9.8) | 3.0 (1.0, 9.0) | 0.657 |

Values are presented as mean±SD for age and median (IQR) for BMI, parity, and years postmenopause. P values were calculated by t-test (age) and Mann–Whitney U tests (others).
